# Survey of Microcystin-Producing Cyanobacteria in French Lakes of Various Trophic Status Using Environmental and Cyanobacterial Parameters and an Active Mussel Biomonitoring

**DOI:** 10.3390/toxins17050245

**Published:** 2025-05-15

**Authors:** Emilie Lance, Alexandra Lepoutre, Luc Brient, Nicolas Maurin, Emmanuel Guillon, Alain Geffard, Dominique Amon-Moreau

**Affiliations:** 1 UMR-I 02 INERIS-URCA-ULH Stress Environnementaux et Biosurveillance des Milieux Aquatiques, Bâtiment 18, BP 1039, 51687 Reims CEDEX 2, Francealain.geffard@univ-reims.fr (A.G.); 2 UMR ECOBIO, Université de Rennes 1, 35042 Rennes CEDEX, France; 3 Institut de Chimie Moléculaire Reims—UMR CNRS 7312, UFR Sciences Exactes et Naturelles, Bâtiment 18, BP 1039, 51687 Reims CEDEX 2, France; nicolas.maurin@univ-reims.fr (N.M.); emmanuel.guillon@univ-reims.fr (E.G.); 4 Établissement Public Territorial de Bassin Seine Grands Lacs, 12 rue Villiot, 75012 Paris, France; dominique.amon-moreau@onf.fr

**Keywords:** cyanobacteria, microcystins, monitoring, bivalves, biomonitoring

## Abstract

Microcystins (MCs), hepatotoxins produced by cyanobacteria, represent a potential threat to aquatic ecosystems and human health. Measuring various environmental and cyanobacterial parameters in water samples can be useful for monitoring water quality and assessing risk but remains a short-term approach. Beyond local risk assessments, estimating global and medium-term levels of freshwater contamination by MC-producing cyanobacteria is challenging in large lakes due to the spatio-temporal variability of their proliferation and the need to multiply sampling dates and locations. In such conditions, a sentinel organism can be valuable for monitoring MCs in situ and providing a time-integrated picture of contamination levels at various stations. We previously assessed the ability of the freshwater bivalves *Anodonta anatina* and *Dreissena polymorpha* to act as biointegrators of MCs, even under low exposure levels to cyanobacteria. In this study, through a two-season investigation in several French lakes experiencing moderate cyanobacterial blooms, we evaluated the relevance of various parameters (cyanobacterial density and biovolume, chlorophyll-a, and phycocyanin) as well as the use of bivalves as indicators of medium-term freshwater contamination by MC-producing cyanobacteria. MC concentrations in cyanobacterial biomass (intracellular MCs) and in bivalves (free MCs, being unbound, and total free and protein-bound accumulated MCs) were measured alongside the characterization of phytoplankton communities. Both mussels integrated and highlighted the presence of intracellular MCs in the environment over the period between two successive water samplings, even at low contamination levels, demonstrating their suitability for in situ biomonitoring of MC-producing cyanobacteria. The results are discussed in terms of the strengths and limitations of different parameters for assessing MC contamination levels in waters depending on the objective (managing, preventing, or global evaluation) and the monitoring strategies used.

## 1. Introduction

Freshwater toxic cyanobacteria are a source of growing concern worldwide as their proliferations expanded around the 1980s due to climate warming and eutrophication, leading to ecological and economic disturbances and representing an ecological and sanitary threat [1,2]. Among cyanotoxins are the hepatotoxin microcystins (MCs), nodularins, and cylindrospermopsins and the neurotoxin anatoxin-a and saxitoxins, which provoke chronic or acute intoxications of animals and humans across the globe [3,4]. Here we focused on the most frequent cyanotoxins found in the environment: the hepatotoxin MC produced by several genera such as *Anabaena, Dolichospermum, Leptolyngbya, Microcystis, Nostoc, Phormidium, Planktothrix*, and *Synechococcus*, for which 246 variants have been described so far [5]. MCs are mostly intracellular (intracell) during cyanobacterial proliferations but are released in the medium and dissolved or adsorbed on particles during cyanobacterial senescence. Once in cells of targeted organisms, MC can increase the phosphorylation of cytoskeletal proteins and impair the structure of the cytoskeleton, which can lead to hepatic hemorrhage and death [6]. In cells, MCs interact with the protein phosphatases (PPases) during a two-step mechanism: first through a reversible binding and, after few hours, through a covalent binding, both inactivating PPases [7,8,9]. Thus, two fractions of MC accumulation can be quantified in organisms: (i) ‘free MCs’, unbound in tissues, and (ii) ‘total MCs’, encompassing free and protein-bound MCs [10].

Freshwater bodies subjected to cyanobacterial proliferations require suitable monitoring to identify and minimize the risks towards public health and aquatic biota. Traditional surveys of cyanobacteria and of cyanotoxins are based on visual monitoring coupled with water sampling to determine cyanobacterial cell density or biovolume and, sometimes, to quantify concentrations of cyanotoxins in the phytoplankton biomass (intracell) or/and dissolved in the water (extracellular/extracell) [1]. Cyanobacterial densities can also be indirectly estimated via chlorophyll a (Chl a) quantification or with spectrofluorometric probes measuring phycocyanin (PC) fluorescence [11,12], but these approaches do not provide information about the cyanotoxins produced.

Multi-site monthly monitoring can also be applied to characterize the overall contamination levels of an aquatic ecosystem. However, ecosystem-scale monitoring of cyanotoxin concentrations for long-term environmental risk assessment can be uncertain. Indeed, quantifying MCs through discrete (e.g., weekly or monthly) water sampling may not accurately reflect the spatial and temporal dynamics of cyanobacterial populations. Cyanobacterial blooms are not homogenously distributed in water and can be temporally (e.g., rapid proliferations and decays) and spatially (i.e., vertical and horizontal migrations) ‘patchy’ [13,14]. Therefore, developing integrative samplers to monitor MCs or the presence of their producers in aquatic ecosystems is mandatory for a better medium-term insight of water contamination levels by MCs. Passive integrators like Polar Organic Compound Integrative Samplers (POCIS) or Solid Phase Adsorption Toxin Tracking (SPATT) were developed to follow-up dissolved MCs in fresh waters [15,16,17]. However, rapid clogging and saturation phenomena can affect their accumulation kinetics [18]. Moreover, POCIS and SPATT only integrate dissolved compounds and are not suitable to highlight the presence of intracellular cyanotoxins.

As filter-feeders with high filtration rates and accumulation capacities, bivalves are used as integrators of various pollutants (e.g., metals, microplastics, organochlorine contaminants, and parasites) in biomonitoring studies [19,20,21,22]. Some bivalves have already been proposed as biomonitoring tools of MC-producing cyanobacteria in water in relation to their ability to reveal the presence of intracellular MCs when exposed to MC-producing cyanobacteria in a laboratory or in situ [17,23,24,25,26]. Freshwater bivalves ingest cyanobacteria and MCs accumulate in free and total (free plus protein-bound) fractions in their tissues [17,25,26,27,28]. Laboratory exposures of the species *Anodonta anatina* and *Dreissena polymorpha* to various densities of a MC-producing cyanobacteria showed that both bivalves could be used as bioindicators of toxigenic cyanobacteria in fresh waters. *A. anatina* rapidly (from day 1 of exposure) revealed low densities of MC-producing cyanobacteria (equivalent to 1 µg intracell MC/L) and highlighted a previous occurrence due to its slow MC elimination after the end of exposure [26]. *D. polymorpha* allowed an estimation of intracellular MC concentration levels in water and highlighted their dynamics (proliferation and end of proliferation) in relation to the fast free MC elimination from tissues after exposure [26].

The choice of which accumulation fraction (free or total MCs) to quantify during a biosurvey depends on its dynamics within the bioindicator’s tissues, as well as on the ease and reliability of its analysis. At a low environmental contamination level (1 µg intracell MC/L), free MCs in *D. polymorpha* tissues were detected only after 3 days, whereas total MCs were detected at the first day of exposure [26]. The total MC fraction encompasses the protein-bound fraction that may be predominant, but is slowly eliminated from tissues, including after 21 days of depuration [26]. Therefore, the quantification of total MCs in bivalve tissues may be a more sensitive tool to detect MC-producing cyanobacteria in waters but may less faithfully represent the dynamic of cyanobacteria in the medium. On the other hand, free MCs further reflected more rapidly and efficiently the MC dynamics for both *A. anatina* and *D. polymorpha* in relation with their rapid elimination from tissues post exposure. The results previously obtained in the laboratory with a planktonic cyanobacteria species (*Planktothrix* sp.) [26] still needed to be validated in situ under conditions involving multi-genus, multi-species, and multi-genotype cyanobacterial proliferations, including with low intracellular MC concentrations (1 µg intracell MC/L). In this study, mussels (*A. anatina* and *D. polymorpha*) were caged at multiple sites across three French lakes (the Ailette, Der, and Temple lakes), which are artificial reservoirs constructed for recreational purposes or for regulating the flow of the Seine River and which experience recurring annual cyanobacterial blooms.

Nine stations with contrasting hydrological conditions and cyanobacterial bloom intensities were investigated during the summers of 2016, 2017, and 2018. For each sampling, environmental intracellular MCs, as well as free and total MCs in mussels, were quantified. In addition, we monitored the composition of the phytoplankton community and measured Chl a and PC concentrations in the water. The results are discussed in terms of the effectiveness of various parameters (Chl a, PC, and cyanobacterial density or biovolume) and of an integrative monitoring strategy using freshwater mussels to detect the presence of MC-producing cyanobacteria. Potential approaches to improving long-term cyanotoxin biosurvey are also considered.

## 2. Results

### 2.1. Cyanobacterial Community Composition, Densities, and Biovolumes

#### 2.1.1. Ailette Lake

**Figure 1:**
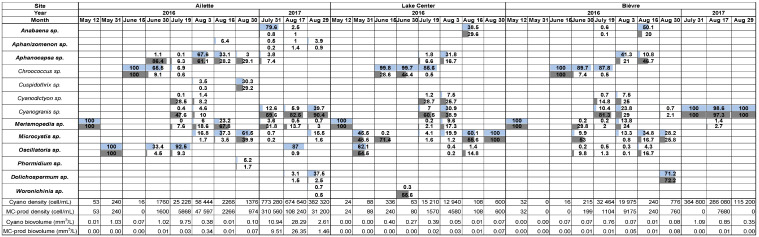
Proportional biovolumes (in light blue) and density (in light grey) of cyanobacteria at different sites of the Ailette Lake in summer periods 2016 and 2017. Data regarding MC producing cyanobacteria are presented in bold.

Among phytoplanktonic communities, cyanobacteria dominated during the summer months of 2017 and 2018 (see Supplementary Materials Figures S1 and S2). From May to August 2016, the cyanobacteria genera with the highest frequency of occurrence (FO, number of times the species was observed related to the total number of samplings) varied between sites: *Merismopedia*, *Microcystis*, and *Oscillatoria* (FO 50%) in Bièvre, *Microcystis* (FO 75%) in the lake center, and *Aphanocapsa* (FO of 62.5%) in Ailette. Most of these dominating genera were potent MC producers (Figure 1). MC-producing cyanobacteria represented from 4.5% (mid-June in the lake center) to 100% of total cyanobacterial biovolumes (12 May at all sites) in 2016 (Figure 1).

During July and August 2017 in Bièvre, the genus with the highest FO was the picocyanobacterium *Cyanogranis* (100%). In Ailette, the genera with the highest FO were *Aphanizomenon* (100%), *Cyanogranis* (100%), and *Merismopedia* (100%), which were present at all sampling dates. MC-producing cyanobacteria were observed only once in Bièvre on 17 August and consisted of *Merismopedia*, a picocyanobacteria which represented 1.4% of cyanobacterial biovolume at that date. In Ailette in 2017, MC-producing cyanobacteria represented from 56.5% (29 August) to 93.1% (17 August) of cyanobacterial biovolumes (Figure 1).

In 2016, in Bièvre (19 July) and Ailette (19 July and 3 August), the total cyanobacteria densities (Figure 1) exceeded the former WHO alert level 1 for water quality (20,000 cell/mL), which is the threshold at which cyanotoxin quantification is recommended [1]. MC-producing cyanobacteria surpassed this threshold only in Ailette on 19 July (22,080 cell/mL). Due to the predominance of picocyanobacteria, the biovolumes of both total and MC-producing cyanobacteria (Figure 1) remained below the revised WHO alert level 1 for recreational waters (i.e., 4 mm^3^/L) [1].

In 2017, cyanobacterial densities exceeded the former WHO water quality guideline level 2 (100,000 cell/mL) at all sites and on all sampling dates, representing a potential health hazard [29]. However, notable differences were observed between sites regarding the densities of MC-producing cyanobacteria: in Bièvre, MC-producing cyanobacteria were not detected on 31 July and 29 August and remained below the 100,000 cell/mL threshold on 17 August (7680 cell/mL). In contrast, in Ailette, MC-producing cyanobacteria densities exceeded 100,000 cell/mL on 31 July and 17 August. The more recent WHO level 1 threshold based on cyanobacterial biovolume in recreational waters (4 mm^3^/L) was exceeded in Ailette during both summer months, but not in Bièvre (Figure 1). The level 2 threshold (above 8 mm^3^/L of cyanobacteria) was exceeded in Ailette in late July and mid-August, both if considering all cyanobacteria genera (10.94 and 28.29 mm^3^/L) or only MC-producing cyanobacteria (9.51 and 26.35 mm^3^/L).

In 2016, disparities were observed between the percentages of MC-producing cyanobacteria among total cyanobacteria when considering cell densities versus biovolumes. For instance, cell densities revealed a dominance of MC-producing cyanobacteria in mid-June in the lake center and in late June in Ailette (71.4 and 90.9% of cells, respectively). However, at the same dates, the biovolume of MC-producing cyanobacteria represented less than 5 and 24.7% of the total biomass, respectively. On the opposite, in early August in Bièvre and the lake center, MC-producing genera were less abundant (representing respectively 49.5 and 35.4% of cell densities) but dominated the overall cyanobacterial biomass, with biovolumes representing, respectively, 68.7 and 61.7% of the cyanobacterial biomass. However, in 54% of samples, the percentages of MC-producing cyanobacteria in density and biovolume were similar.

In 2017, cyanobacterial densities and biovolumes in Bièvre were dominated by non-MC-producing cyanobacteria (Figure 1). However, in Ailette, MC-producing cyanobacteria represented less than 50% of cyanobacterial densities but from 55.8 to 93.1% of cyanobacterial biovolumes.

#### 2.1.2. Der Lake

**Figure 2:**
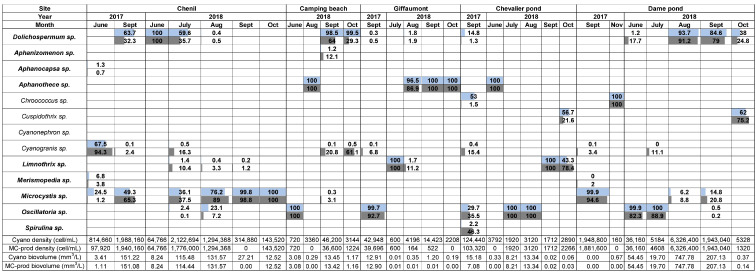
Proportional biovolumes (light blue) and cell densities (light grey) of cyanobacteria at different sites of Lake Der during the summer periods of 2017 and 2018. Data related to MC-producing cyanobacteria are highlighted in bold.

At the sites of the Der Lake, data showed a combination of non-MC-producing cyanobacteria such as *Cyanogranis* and *Chroococcus* and of MC-producing cyanobacteria such as *Microcystis*, *Oscillatoria*, and *Dolichospermum.* A total of 13 genera were identified at all sites; the most prevalent were *Dolichospermum*, *Oscillatoria*, and *Microcystis.* As shown in Figure 2, genera including species and strains capable of producing MCs were largely predominant at all sites during both 2017 and 2018. The cyanobacterial biomass reached a high cellular density in summer 2017, particularly in September in the La Dame and Chenil ponds, with concentrations approaching 2 million cells/mL. In the Chenil station in July 2018, up to five cyanobacterial genera were co-proliferating simultaneously, whereas on other sampling dates and at other sites, individual genera often dominated (e.g., more than 90% of density or biovolume for *Dolichospermum*, *Aphanothece*, or *Oscillatoria*). In June 2017, 94.3% of the cyanobacterial density in the Chenil pond was represented by the picocyanobacterium *Cyanogranis*, which nonetheless only represented 67.5% of the total cyanobacterial biovolume. When larger cyanobacteria proliferated, such as *Microcystis* (in Chenil in September 2017 and from August to October 2018, as well as in La Dame in September 2017), the proportional density and biovolumes were high (100%) and equivalent, both largely exceeding the alert level 2 (8 mm^3^/L) of the WHO water quality guidelines. During the summer months of 2017 and 2018, the Chenil pond was dominated by MC producers (*Dolichospermum* and *Microcystis*), with biovolumes reaching 151 mm^3^/L in September 2017 and 131 mm^3^/L in August 2018—both largely exceeding the level 2 threshold for recreational waters. The level 2 threshold of MC-producing cyanobacterial biovolumes was also exceeded in September 2018 at the site Camping beach (13.4 mm^3^/L of cyanobacteria with 98.5% of *Dolichospermum*), in September 2017 at Giffaumont (12.9 mm^3^/L of cyanobacteria with 99.7% of *Oscillatoria*), in July and August 2018 at Chevalier (8.2 and 13.3 mm^3^/L of cyanobacteria with 100% of *Oscillatoria*), and continuously from June to September 2018 at the Dame pond (ranging from 19.7 to 747.8 mm^3^/L of cyanobacteria with 100% of *Oscillatoria* or from 84.6 to 93.7% of *Dolichospermum*).

#### 2.1.3. Temple Lake

**Figure 3:**
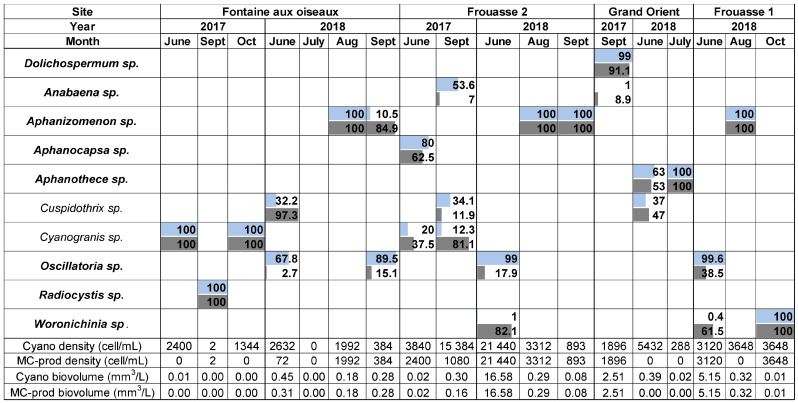
Proportional biovolumes (light blue) and cell densities (light grey) of cyanobacteria at different sites of Temple Lake during the summer periods of 2017 and 2018. Data related to MC-producing cyanobacteria are shown in bold.

In 2017 and 2018, the stations of the Temple Lake exhibited much lower cyanobacterial densities compared to those of the Der Lake, with a maximum of 21,444 cell/mL recorded in Frouasse 2 in June 2018 (Figure 3). The cyanobacterial communities were poorly diversified (10 genera in total), with recurrent dominance by a single genus—accounting for more than 80% of the cell density or biovolume—in 14 out of the 18 samplings, regardless of site or month. For example, *Cyanogranis* dominated (100% of density and biovolume) at the site Fontaine aux Oiseaux in both June and October 2017, while *Aphanizomenon* dominated (100% of density and biovolume) at the sites Fontaine aux Oiseaux, Frouasse 1, and Frouasse 2 over a period of four months. Frouasse 2 was the only site where the biovolume of MC-producing cyanobacteria exceeded the level 2 threshold (8 mm^3^/L) for recreational waters, with a value of 16.8 mm^3^/L recorded in one month. The first WHO alert level for recreational waters (i.e., 4 mm^3^/L) was also exceeded once at Frouasse 1 in June 2018, with the biovolume of MC-producing cyanobacteria reaching 5.15 mm^3^/L.

### 2.2. MC Measurement in Cyanobacterial Biomass and in Bivalve Tissues

#### 2.2.1. Ailette Lake

**Figure 4:**
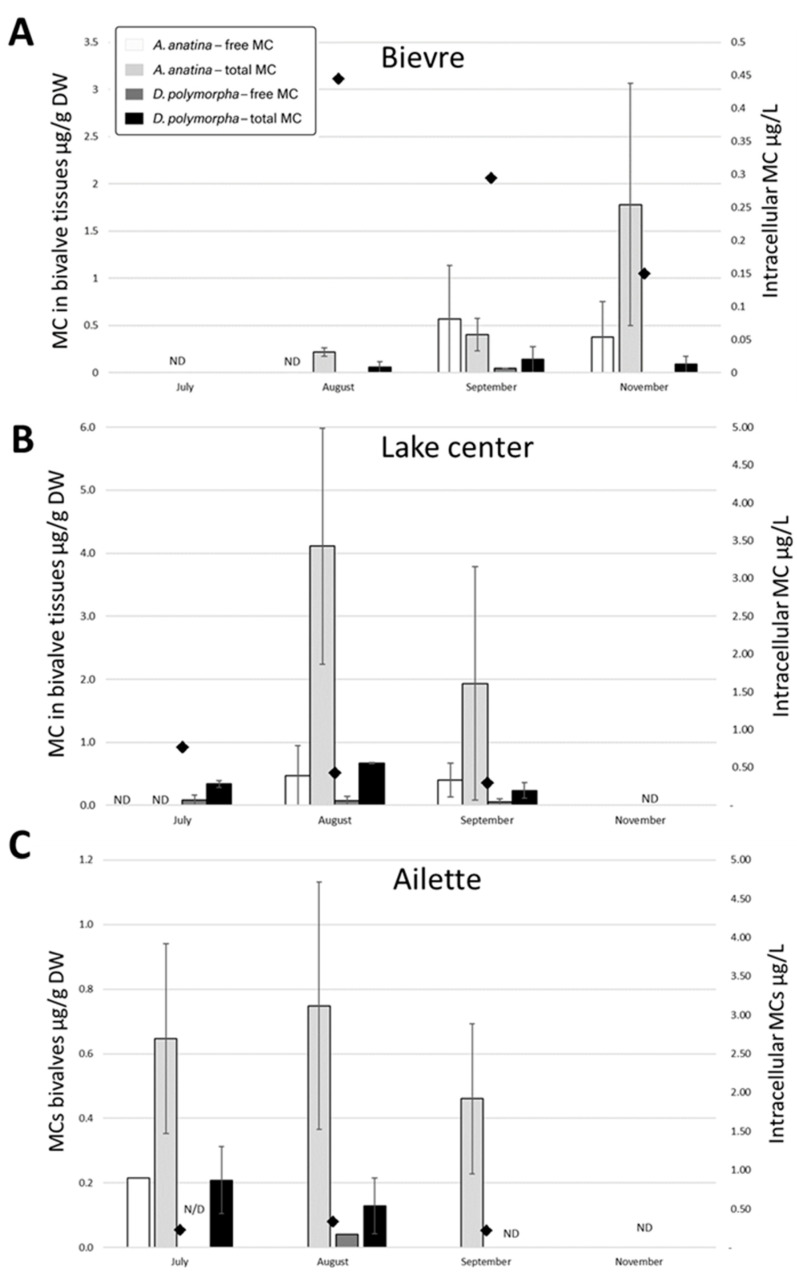
Accumulation (µg/g DW) of free (white bars) and total (light grey bars) MC in *A. anatina* (AN) and of free (dark grey bars) and total (black bars) MC in *D. polymorpha* (DP), caged from May to November 2016 in Bièvre (**A**), lake center (**B**), and Ailette (**C**). Intracellular MC concentrations in water are represented by black diamonds. ND indicates that the analysis was not performed.

In 2016, MC concentrations in the water remained low across all sites, ranging from 0.22 µg to 1.07 µg intracell MC/L (Figure 4). However, despite these low levels, MCs were continuously detected in Ailette and the lake center from July to September. In Bièvre, MCs were detected in August and September but not in November. Both free and total MCs were detected in the two bivalve species during 2016. However, possibly due to their inability to escape unfavorable conditions, *D. polymorpha* died in September 2016 in Ailette, coinciding with a large accumulation of sediment in the cages.

The highest free and total MC concentrations were observed in *A. anatina*: free MCs reached 0.57 µg/g DW in Bièvre in September and total MCs peaked at 4.11 ± 1.87 µg/g DW in the site lake center in August. Overall, *A. anatina* exhibited significantly higher concentrations of both free and total MCs compared to *D. polymorpha* (Mann–Whitney test, *p* < 0.01). Furthermore, the frequency of occurrence (FO) of MCs, regardless of fraction, was slightly higher in *A. anatina* (54% of samples) than in *D. polymorpha* (42% of samples), particularly at low MC concentrations in tissues. For instance, in November in Bièvre, free MCs were detected in the digestive glands of *A. anatina* but not in whole *D. polymorpha* specimens.

When comparing the two accumulation fractions in bivalves throughout the 2016 monitoring period, total MCs were detected at significantly higher concentrations than free MCs: from 0 to 2.10 ± 0.07 µg/g DW for total MCs vs. 0 to 0.29 ± 0.11 µg/g DW for free MCs in *D. polymorpha*, and from 0 to 7.88 ± 2.40 µg/g DW for total MCs vs. 0 to 1.78 ± 0.05 µg/g DW for free MCs in *A. anatina* (Mann–Whitney test, *p* < 0.01). The comparison of detection frequencies for each fraction showed that free MCs were found in 29.4% of *D. polymorpha* samples and 20.7% of *A. anatina* digestive gland samples, whereas total MCs were detected in 65.2% and 86.8% of *D. polymorpha* and *A. anatina* samples, respectively.

**Figure 5:**
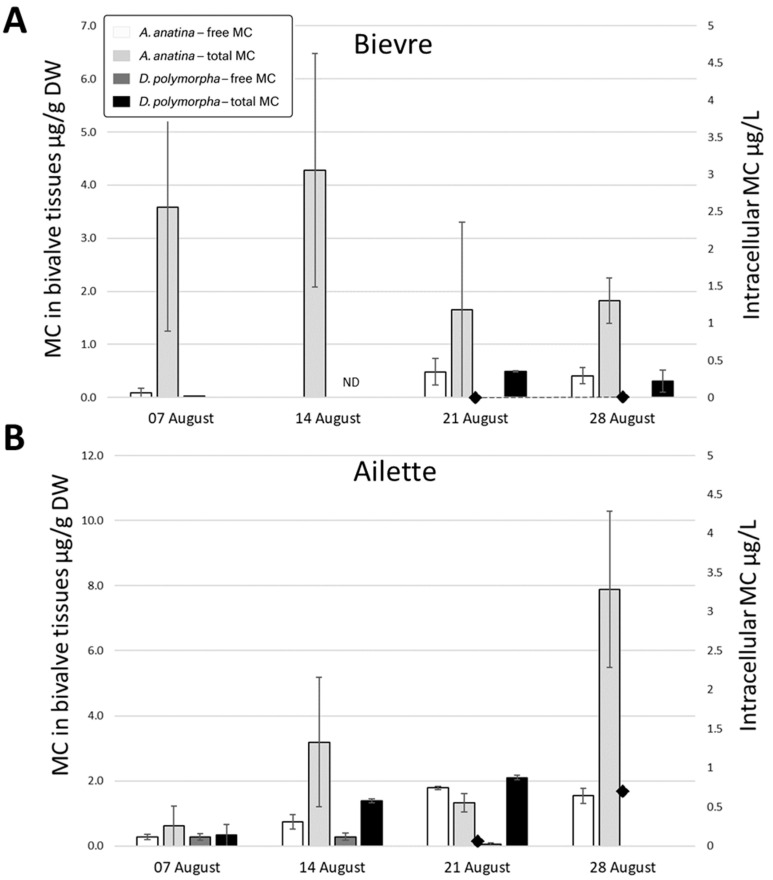
Accumulation (µg/g DW) of free (white columns) and total (light grey columns) MCs in *A. anatina* (AN) and of free (dark grey columns) and total (black columns) MCs in *D. polymorpha* (DP) caged in August 2017 in Bièvre (**A**) and Ailette (**B**) sites. Intracellular MC concentrations in water are represented by black diamonds (no data available on 7 and 14 August). ND indicates that the analysis was not performed.

Data on MC concentrations in the water were not available for the first two weeks of August 2017. Subsequently, until the end of August, no MCs were detected in Bièvre. In Ailette, environmental MC concentrations remained below 1 µg intracellular MC/L, with a maximum of 0.7 ± 0.04 µg/L recorded on 28 August (Figure 5). MCs were detected in 58% of mussel tissue samples, regardless of the accumulation fraction or species. Notably, MCs were detected in 44% of mussels caged in Bièvre, despite the absence of detection in the corresponding phytoplankton samples.

As observed in 2016, *A. anatina* showed significantly higher concentrations of both free and total MCs compared to *D. polymorpha* for the same fractions (Mann–Whitney test, *p* < 0.01). The comparison of accumulation fractions further revealed that total MC concentrations were significantly higher than free MC concentrations in both species (Mann–Whitney test, *p* < 0.01). Regarding detection frequency in August 2017, free MCs were found in 25% of whole *D. polymorpha* samples and in 66.7% of *A. anatina* digestive gland samples. Total MCs were detected in 46.9% of *D. polymorpha* samples and in 89.3% of *A. anatina* samples.

#### 2.2.2. Der Lake

**Figure 6:**
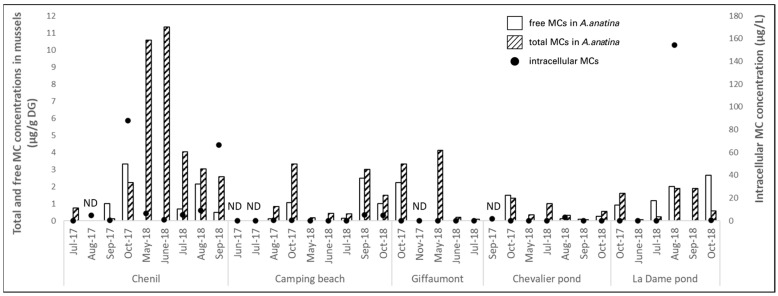
Accumulation (µg/g DW) of free (white columns) and total (hatched columns) microcystins (MCs) in *A. anatina* caged at various sites of Der Lake. Intracellular MC concentrations in water are shown as black dots. ND indicates that the analysis was not performed.

Intracellular MC concentrations in the water were high at the Chenil site during the summer periods of 2017 and 2018, ranging from 0.19 to 88.10 µg intracellular MC/L, with a mean of 20.15 µg/L (Figure 6). The maximum concentration (88.10 µg/L) was recorded in October 2017, coinciding with a complete dominance (100%) of *Microcystis* in the cyanobacterial community and cell densities reaching 1,934,400 cells/mL. Bivalve tissues were more contaminated by total MCs (free and protein-bound) in October 2017 than in previous months, with a mean concentration of 2.23 µg/g FW in digestive glands. In May and June 2018, MC concentrations in mussel tissues at the Chenil site were higher than in 2017 (up to 11.3 µg/g FW DG), despite the lower environmental concentrations revealed by the two monthly water samplings (Figure 6).

At the La Dame pond, four out of nine water samples tested positive for MCs during 2017 and 2018, with concentrations ranging from 0.29 to 154.51 µg intracellular MC/L. Concentrations exceeded 0.5 µg/L only in the September and October 2018 samplings. In contrast, total or free MCs were consistently detected in mussel tissues at this site, with concentrations ranging from 0.24 to 2.65 µg total MCs/g DW.

At the site Camping Beach, Giffaumont, and the Chevalier pond, intracellular MC concentrations in water were low—ranging from 0.02 to 5.36 µg/L, with mean values of 1.21, 0.12, and 0.69 µg/L respectively—reflecting the low densities of MC-producing cyanobacteria observed at these sites (Figure 6). At the site Camping Beach, four out of nine water samples tested positive for MCs, while total MCs were detected in seven out of nine mussel tissue samples (and in six out of nine for free MCs). Similarly, in Giffaumont and Chevalier, the frequency of MC-positive samples was higher in mussel tissues (four out of five and six out of seven for total MC, respectively) than in water samples (two out of five and three out of seven, respectively).

Overall, intracellular MC concentrations in the environment often exceeded the WHO health threshold for recreational waters, particularly at the Chenil and La Dame sites and to a lesser extent at the site Camping Beach (approximately 5 µg/L in September and October 2018), where recreational activities such as swimming take place.

With regard to the biosurvey, free and total MCs were detected in the digestive glands of *A. anatina* during both 2017 and 2018 at all sites (Figure 6). The highest free and total MC concentrations in *A. anatina* were observed at the Chenil site: 3.31 µg/g DW for free MCs in October 2017 and 11.35 µg/g DW for total MCs in June 2018. Across the two-year campaign, total MCs in *A. anatina* were detected at significantly higher frequencies and concentrations than free MCs (31 out of 32 samples and from 0.10 to 11.35 µg/g DW for total MCs vs. 19 out of 25 samples and from 0.13 to 3.30 µg/g DW for free MCs) (Mann–Whitney test, *p* < 0.01).

#### 2.2.3. Temple Lake

**Figure 7:**
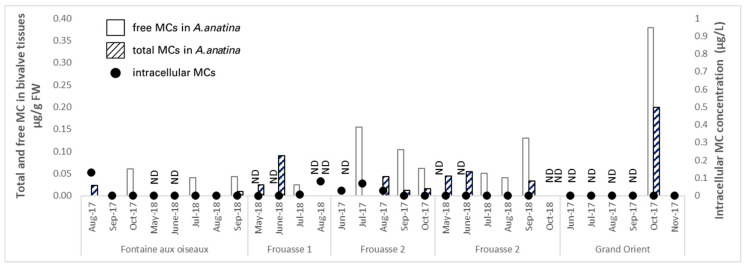
Accumulation (µg/g DW) of free (grey columns) and total (hatched columns) MCs in *A. anatina* caged at sites of the Temple Lake. Intracellular MC concentrations in water are shown as black dots. ND = not determined.

The sites of Temple Lake were less contaminated by intracellular MCs than those of Der Lake. Some sites dried out during the summer season (indicated as ND in Figure 7). Intracellular MCs were quantified in only one out of eight water samples at Fontaine aux Oiseaux, with a concentration of 0.13 µg intracellular MC/L (Figure 7). At this site, MCs were detected in bivalve tissues on three occasions (August and October 2017, and September 2018), but at very low concentrations (ranging from 0.02 to 0.06 µg free MC/g DW).

The Frouasse 1 site was also weakly contaminated by MCs, with a maximum of 0.08 µg intracellular MC/L in water and 0.09 µg free MC/g DW in mussel tissues. Monitoring at the Grand Orient site began in October 2017; although no intracellular MCs were detected in water, mussels showed MC contamination in tissues of up to 0.38 µg free MC/g DW. This site was dry during the 2018 campaign, and no data are available for that period.

At Frouasse 2, four out of nine water samples tested positive for MCs, with a maximum concentration of 1.02 µg intracellular MC/L in October 2018. The integrative monitoring using caged mussels more frequently revealed MC contamination than monthly water samplings, with six out of seven mussel samples showing the presence of free or total MCs and concentrations reaching up to 0.15 µg free MC/g DW.

### 2.3. Correlations Between Environmental Parameters, MC Concentrations in Water and in Mussels, and Water Contamination by Cyanobacteria

#### 2.3.1. Ailette Lake

Overall, during the two-year monitoring campaign at Ailette Lake, intracellular MC concentrations did not correlate with chlorophyll a (Chl a), phycocyanin (PC), cyanobacterial cell densities, total cyanobacterial biovolumes, or biovolumes of MC-producing cyanobacteria (see data in the Supplementary Table S1). No significant correlations were observed either with free or total MC concentrations in whole *D. polymorpha* or in the digestive glands of *A. anatina* when considering the same sampling dates. However, a significant correlation was found between densities of MC-producing cyanobacteria and free MC concentrations in whole *D. polymorpha* (Pearson’s r = 0.89, *p* < 0.01).

Over the two years, biovolumes of MC-producing cyanobacteria were strongly correlated with their cell densities (Pearson’s r = 0.98, *p* < 0.01), with overall cyanobacterial densities (r = 0.84, *p* < 0.01), and with total cyanobacterial biovolumes (r = 0.99, *p* < 0.01). Chl a did not show significant correlations with any of these parameters. In contrast, PC concentrations were significantly correlated with total cyanobacterial densities (r = 0.91, *p* < 0.01), MC-producing cyanobacteria densities (r = 0.89, *p* < 0.01), and their biovolumes (r = 0.78, *p* < 0.05).

#### 2.3.2. Der and Temple Lake

During the 2017 and 2018 study in the Der and Temple Lakes, intracellular MC concentrations did not correlate with Chl a or PC (see data in the Supplementary Table S1), nor with free or total MC concentrations in the tissues of *A. anatina* (considering the same sampling dates). However, intracellular MC concentrations were significantly correlated with total cyanobacterial densities (r = 0.86, *p* < 0.01) and biovolumes (r = 0.84, *p* < 0.01), as well as with densities (r = 0.84, *p* < 0.01) and biovolumes (r = 0.87, *p* < 0.01) of MC-producing cyanobacteria. Over the two years, biovolumes of MC-producing cyanobacteria correlated strongly with overall cyanobacterial densities and biovolumes (Pearson’s r = 0.99 and 1.00 respectively, *p* < 0.01) and with the densities of MC-producing cyanobacteria (Pearson’s r = 0.99, *p* < 0.01). Chl a did not correlate with any of these parameters (all Pearson’s r < 0.49), whereas PC concentrations were significantly correlated with overall cyanobacterial densities (Pearson’s r = 0.66, *p* < 0.05), MC-producing cyanobacteria densities (r = 0.66, *p* < 0.05), and their biovolumes (Pearson’s r = 0.71, *p* < 0.01).

When separating data by contamination levels, we observed that correlations between intracellular MC concentrations and the biovolumes of total or MC-producing cyanobacteria became significant only above a biovolume threshold of approximately 40 mm^3^/L. For cyanobacterial biovolumes < 40 mm^3^/L, no correlation was observed between intracellular MC and the biovolume of total (R^2^ = 0.15) or MC-producing (R^2^ = 0.20) cyanobacteria. For biovolumes ranging from 40 to 250 mm^3^/L, these correlations became significant (R^2^ = 0.66 and 0.76 for total and MC-producing cyanobacteria, respectively, *p* < 0.05) and were even stronger for biovolumes between 250 and 800 mm^3^/L (R^2^ = 0.98 for both total and MC-producing cyanobacteria, *p* < 0.01).

Similarly, correlations between MCs accumulated by mussels (free and total fractions) and total cyanobacterial densities (r = 1.00 and 0.79 for free and total fractions respectively, *p* < 0.01), or biovolumes (r = 1.00 and 0.71, *p* < 0.01 and <0.05, respectively), as well as with MC-producing cyanobacteria densities (r = 0.98 and 0.63, *p* < 0.01 and <0.05), and their biovolumes (r = 0.96 and 0.75, *p* < 0.01 and <0.05), became significant when intracellular MC concentrations in water exceeded 1 µg/L.

**Figure 8:**
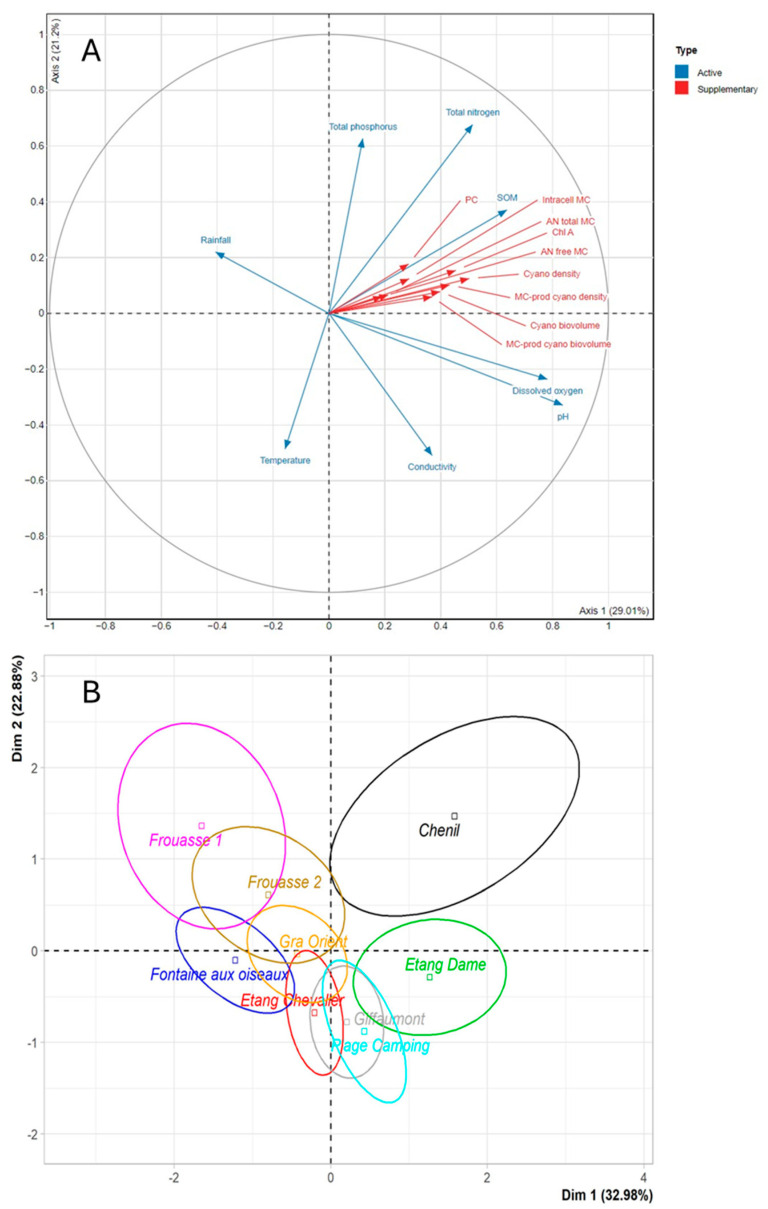
Principal Component Analysis (PCA) with (**A**) environmental variables (rainfall n = 63; temperature n = 63; pH n = 63; dissolved oxygen n = 63; total phosphorus n = 63; total nitrogen n = 63; conductivity n = 63; SOM n = 62), phytoplanktonic or cyanobacterial parameters (Chl a n = 53; PC n = 52; total density “cyano density” n = 59; total biovolume “cyano biovolume” n = 59; MC-prod cyanobacteria density n = 59; MC-prod cyanobacteria biovolume n = 59; intracellular MC “Intracell MC” n = 82), and mussel *A. anatina* parameters (free accumulated MC “AN free MC” n = 28; total accumulated MC “AN total MC” n = 40) measured during the 2017 and 2018 campaigns and (**B**) the sites of the Der (Chenil, La Dame, Plage Camping, Giffaumont, and Etang Chevalier) and Temple (Frouasse 1 and 2, Grand Orient, and Fontaine aux Oiseaux) Lakes.

The first principal component (Dim 1) of the Principal Component Analysis (PCA) explained 29.01% of the variance, while the second component (Dim 2) explained 22.2%, together accounting for 51.21% of the total variance. Variables related to cyanobacteria (e.g., intracellular MCs and total and MC-producing cyanobacterial densities and biovolumes) were oriented toward the positive side of both Dim 1 and Dim 2, suggesting strong correlations among these biological metrics (Figure 8). These variables also showed strong correlations with PC and Chl a, as well as with free and total MC contents in mussel tissues. Total nitrogen and total phosphorus were also oriented toward the positive side of Dim 1, indicating an association with increased cyanobacterial abundance and environmental MC production.

In the PCA plot including sampling locations, clusters revealed similarities among sites based on their environmental and biological data. The PCA confirmed that Der Lake was more contaminated by cyanobacteria and MCs than Temple Lake (distinguished along principal component 1), particularly at the Chenil and La Dame sites. These sites formed distinct clusters compared to others, sharing high values of intracellular MCs, cyanobacterial densities and biovolumes, Chl a, PC, and suspended organic matter (SOM). In particular, the Chenil site, located on the positive side of Dim 1, showed elevated nutrient levels (nitrogen and phosphorus) associated with high densities and biovolumes of cyanobacteria (both total and MC-producing), as well as high MC concentrations in water (intracellular) and in mussel tissues (accumulated) (Figure 8).

In contrast, sites such as Frouasse 1, Fontaine aux Oiseaux, and Frouasse 2, located on the negative side of Dim 1, exhibited different environmental characteristics (e.g., higher rainfall, lower conductivity, and lower pH) associated with lower cyanobacterial abundance and MC levels (Figure 8). 

## 3. Discussion

Determining the overall level of water contamination by cyanotoxins at different sites within a water body could help water managers implement targeted preventive measures, thereby reducing costs and increasing the effectiveness of interventions. Cyanobacterial blooms often exhibit significant spatiotemporal variability, influenced by various factors such as species physiology, hydrological parameters, weather and climate conditions [30]. As a result, monitoring the densities of toxin-producing cyanobacteria or cyanotoxin concentrations in water can be challenging [31]. In this study, we evaluated in situ the potential use of freshwater mussels as integrators and bioindicators of freshwater contamination by MCs by comparing toxin concentrations in their tissues with environmental parameters describing the cyanobacterial community.

### 3.1. Environmental and Cyanobacterial Parameters

In 2016 and 2017, water was sampled in Lake Ailette (referred to as the “lake center”) and in its two tails (“Bièvre” and “Ailette”). Overall, in 2016, cyanobacteria were more predominant in the two tails compared to the main body of the lake. This could be attributed to a combination of factors, including differences in hydrological conditions and water use regulations. Indeed, the main lake is deeper than the two tails (maximum depth of 6.5 m vs. 3 m). During summer, the shallow tails can become hydrologically isolated, resulting in longer water retention times and the formation of thermal stratification. These conditions favor increased water temperatures and promote cyanobacterial proliferations. [1,32]. Moreover, fishing is only permitted in the Ailette and Bièvre tails and is prohibited in the main lake. The combination of catch-and-release fishing practices and the use of bait—known to contribute to eutrophication—likely also plays a role in promoting cyanobacterial development in these two shallow areas [33]. Additionally, while the succession of cyanobacterial species was similar in 2016 and 2017 at the Ailette site, the cyanobacterial species richness decreased at the Bièvre site. This decline may be attributed to variations in physicochemical parameters, which likely induced competitive interactions among taxa in Bièvre, as demonstrated in an in situ study [34].

Measurements of Chl a and PC concentrations are often used to monitor the presence of cyanobacteria [35,36,37,38]. In the two-year study conducted at Lake Ailette, Chl a concentrations in the water were not correlated with cyanobacterial densities or biovolumes. This lack of correlation could be explained by the presence of other Chl a-producing organisms within the phytoplanktonic community, such as Chlorophyceae, as well as by the varying levels of Chl a production among different cyanobacterial genera and species. Moreover, picocyanobacteria dominated on several sampling dates, which strongly reduced the overall cyanobacterial biovolume. As the enumeration of picocyanobacteria can be challenging and potentially underestimated using conventional microscopic methods, their contribution may not be fully reflected in biovolume estimates [39] and a numeration bias may prevent a good correlation between densities, biovolumes, and Chl a. We could expect that Chl a and cell densities and biovolumes would be correlated if bigger cyanobacterial species (such as *Microcystis* sp. or *Planktothrix* sp.) dominated the phytoplankton. For instance, Chl a concentrations and cyanobacteria biovolumes were correlated in Sulejow Reservoir (Poland) when *M. aeruginosa* dominated the phytoplankton communities [40]. However, at all sites, PC concentrations were correlated with total cyanobacterial densities and biovolumes, and only in Der and Temple Lakes where they also correlated with the densities and biovolumes of MC-producing cyanobacteria. Therefore, in this context, PC concentration proved to be a more relevant indicator for evaluating cyanobacterial density.

Some studies have reported correlations between Chl a concentrations and intracellular MC during *Microcystis aeruginosa* blooms, for instance in Poland [40]. However, in the sites of the Ailette, Der and Temple Lakes, intracellular MC concentrations did not correlate with estimated PC or Chl a concentrations. As for Chl a, the lack of such correlation could be attributed to the variability in MC production by cyanobacteria. Indeed, not all cyanobacterial species can produce MC and, among producers, the toxin synthesis varies among genotypes and environmental conditions [41,42]. Therefore, a significant correlation between intracellular MC and Chl a or PC can be expected when only few MC-producing strains dominate the cyanobacterial population with high densities [43]. In Lake Ailette, intracellular MC concentrations remained low (below 1 µg MC/L), even though genera and species known to produce MCs dominated the cyanobacterial community in terms of density (in 50% of samples across all sites in 2016 and 2017) and biovolume (in 60% of samples across all sites in 2016 and 2017).

At the Ailette sites, variations in MC production among cyanobacterial genotypes, combined with the overall low levels of water contamination, may also explain the absence of correlation between intracellular MC concentrations and the densities or biovolumes of potentially MC-producing cyanobacteria. Nevertheless, a correlation between intracellular MCs and cyanobacterial biovolumes has been reported during a *Microcystis* sp. bloom [44], but intracellular MC concentrations were higher than those observed here, ranging from 0.5 µg/L to 120 µg/L in scums.

No parameter was correlated with intracellular MC concentrations in water, particularly when picocyanobacteria dominated the phytoplanktonic communities. For example, the maximum abundances observed in water for the genera *Anabaena* and *Aphanocapsa* were 11.2 × 10^6^ and 4.2 × 10^6^ cells·mL^−1^, respectively, resulting in an *Anabaena/Aphanocapsa* abundance ratio of 2.66. However, after conversion to biovolume, this ratio increased to 131, which is 50 times higher. Therefore, relying solely on cell abundance significantly overestimates the contribution of *Aphanocapsa* relative to *Anabaena* in the analyses performed. Using biovolumes instead of cell abundances provides a more accurate representation of the relationship between MC concentrations in water and cyanobacterial biomass, although this relationship likely becomes meaningful only at higher contamination levels than those observed at the Ailette sites.

The 2017 and 2018 campaign results for the Der and Temple Lakes indicate that there is not always a correlation between the density of MC-producing cyanobacteria and MC concentrations in water. Some stations exhibited low MC levels despite high cyanobacterial densities, which were mainly composed of less toxic picocyanobacteria. This further supports the idea that cell density alone is not a reliable predictor of MC presence. However, some sites in Der Lake—particularly the Chenil and La Dame ponds—showed higher cyanobacterial densities and biovolumes than those recorded at Ailette sites. In these more contaminated sites, correlations between intracellular MC concentrations in water and the biovolume of total or MC-producing cyanobacteria became significant when cyanobacterial biovolumes exceeded 40 mm^3^/L. For biovolumes between 40 and 250 mm^3^/L, the correlations became significant and were even stronger between 250 and 800 mm^3^/L. Therefore, the biovolume of total or MC-producing cyanobacteria can serve as a good predictor of MC presence in the environment, but only when exceeding 40 mm^3^/L—a value well above the WHO level 2 alert threshold for recreational waters (8 mm^3^/L).

At the Temple and Der Lake sites, we examined the overall relationships between physical (e.g., rainfall and surface temperature) and chemical (e.g., nitrate and phosphorus) parameters and variables associated with cyanobacteria (e.g., intracellular MCs and total and MC-producing cyanobacterial densities and biovolumes), as well as MC accumulation in bivalves (free and total fractions). Variables associated with cyanobacteria were strongly correlated with each other and with free and total MC contents in mussel tissues. The PCA confirmed that Der Lake was more heavily contaminated by cyanobacteria and MCs than Temple Lake, particularly at the Chenil and La Dame sites, which also displayed higher nutrient levels (nitrogen and phosphorus). Recent studies have likewise demonstrated a strong association between cyanobacterial abundance, MC concentrations, and nitrogen and total phosphorus levels [45]. It has been suggested that phosphorus often drives initial cyanobacterial growth, whereas ammonium can sustain and enhance bloom duration [46,47]. Therefore, the measurement of both nitrogen and phosphorus can contribute to predicting the potential occurrence of cyanobacterial blooms.

Finally, cyanobacterial biovolume or density—whether of MC-producing taxa or not—does not always provide an accurate estimate of environmental MC contamination. Direct measurements of MCs remain more informative for short-term monitoring. However, new methods need to be developed to better account for the variability in the presence and abundance of MC-producing strains, in order to more reliably assess the medium- to long-term levels of water contamination by MCs.

### 3.2. Information Provided by Mussel Tissues

The caging of bivalves for biomonitoring pollutants in water has advantages over the sampling of native individuals as it allows to (i) control the age, size, and history of contamination of the animals and (ii) compare different sites by reducing potent physiological confounding factors [48,49]. The drawbacks are the potential loss of caging systems and the risk of introducing new species [45]. Here, because *D. polymorpha* is an invasive species, its presence was checked prior to transplantation, as was that of *A. anatina*, even though the latter is non-invasive. In Lake Ailette, a mortality event affecting *D. polymorpha* was observed in September 2016, coinciding with a drop in water level and the presence of large amounts of sediment in half of the cages. In contrast, *A. anatina* survived, likely due to its ability to move and burrow into sediments [46,47].

The nature of the substrate and the potential risk of sediment clogging during water level drops should therefore be taken into account when selecting a species for a biosurvey program. To assess the physiological status of both bivalve models during the caging period, we measured the condition index. The condition index of *D. polymorpha* remained stable throughout the study and was consistent with values previously reported by Kerambrun et al. [50]. In 2016, the condition index of *A. anatina* remained stable across all sites. However, in 2017, it increased at the Ailette site, which likely indicates that the mussels had access to sufficient food resources in the water—supported by the higher cyanobacterial densities and biomass recorded that year. This suggests that cyanobacteria, including MC-producing strains, may have constituted a nutritious food source for the bivalves, as previously demonstrated [51] and despite the concomitant intoxication that they undergo.

In Lake Ailette, although intracellular MC concentrations in phytoplankton remained relatively low (with a maximum of 1.07 µg MC/L), both mussel species were able to reveal the presence of MC-producing cyanobacteria in the water. For example, the digestive glands of *A. anatina* accumulated MCs (4.18 ± 2.20 µg total MC/g DW) on August 14 at the Bièvre site, a date on which neither MC-producing cyanobacteria nor intracellular MCs were detected in the water. A similar observation was reported in Chambers Bay (USA), where MCs were detected in *Mytilus trossulus*, while analyses of phytoplankton biomass alone did not reveal the presence of intracellular MCs [24]. This may be explained by the occurrence of a proliferation of MC-producing cyanobacteria between two water sampling dates, which was subsequently integrated by the mussels. In addition, mussels may also provide information on the presence of MC-producing benthic cyanobacteria, which are not always captured by routine phytoplankton monitoring [51]. Indeed, benthic species such as *Phormidium* sp., detected here, can produce MCs [52,53]. Additionally, recent studies suggest that MCs can be retained in sediments and periodically released back to water [54,55]. As mussels were caged at a depth of 1 m below the water surface in this study, contamination from benthic cyanobacteria or sediment may have occurred during periods of low water levels.

The results obtained here support our previous laboratory study investigating the use of *A. anatina* and *D. polymorpha* as bioindicators of the presence of MC-producing cyanobacteria in their environment. Both mussel species were able to reveal the presence of low MC concentrations in water when exposed to a gradient of a *Planktothrix agardhii* strain producing 1, 10, or 100 µg MC/L [26]. Moreover, the free and total MC concentration ranges (in µg/g DW) quantified in both species caged in Ailette Lake ([0–0.29] free and [0–0.7] total MC in *D. polymorpha*, and [0–1.7] free and [0–7.88] total MC in *A. anatina*) were within the concentration ranges ([0–27] free and [0–1.26] total MC in *D. polymorpha* and [0.10–12.01] free and [0.98–23.38] total MC in *A. anatina*) quantified in mussels exposed in a laboratory to *P. agardhii* producing an equivalent of 1 µg MC/L. This is consistent with the MC concentrations observed in the water at the three investigated stations of the Ailette site, which did not exceed 1 µg MC/L. However, in the laboratory, mussels were subjected to continuous exposure without fluctuations in the density of MC-producing cyanobacteria. In situ, mussels were likely exposed to multiple cyanobacterial proliferations with varying densities of MC-producing cyanobacteria and possible depuration periods in between.

Additionally, in our previous experiment, we monitored cyanobacterial ingestion and MC accumulation in mussels exposed to the filamentous *Planktothrix agardhii*. In the present field study, mussels were exposed to a broader diversity of cyanobacterial morphologies, including picocyanobacteria (*Merismopedia* sp.), colonial species (*Microcystis* sp.), and filamentous cyanobacteria (*Oscillatoria* sp.). Nevertheless, they were able to reveal the contamination of the environment. This suggests that, although these bivalve species are known to sort their food prior to ingestion—based on factors such as particle size and surface chemical properties [56,57]—this sorting does not prevent their use as bioindicator species to monitor various MC-producing cyanobacteria.

As in the laboratory experiment, regardless of sites and years, *A. anatina* accumulated significantly more free and total MCs than *D. polymorpha*. We previously showed that this difference remains regardless of the comparison of MC concentration in a target organ vs. a whole individual (as the digestive glands of *A. anatina* accumulated more MCs than those of *D. polymorpha*) [26]. In this study, free MCs were detected in the tissues of *A. anatina* on several occasions when none were found in *D. polymorpha*. This suggests that *A. anatina* may be more sensitive than *D. polymorpha* for detecting MCs under conditions of low producer density and low MC concentrations in the water (<1 µg/L). Similar findings were reported in a field study using passive sampling with both species in lakes experiencing cyanobacterial blooms, although no direct measurements of MC concentrations in the water were performed in that case [58]. The differences in MC concentrations in tissues of *A. anatina* vs. *D. polymorpha* were attributed to *D. polymorpha’s* selective feeding, its strong MC biotransformation capacities, and the potential ingestion of MC-contaminated sediments by *A. anatina* [58]. However, the follow-up of the ingestion of cyanobacteria by both species and the absence of sediments in the laboratory exposure [26], suggests that the most likely factor would be a more effective detoxification of MCs by *D. polymorpha* compared to *A. anatina*.

As in previous studies [26,28,59,60], total MC concentrations in mussel tissues were higher compared to free MCs. A significant part (i.e., from 50 to 75% during our previous exposures) of MCs in mollusk tissues covalently bind to proteins, among which are PPases. Thus, free MC can be efficiently eliminated from mussel tissues by the detoxification systems involving glutathione [61], whereas the elimination of bound MCs is probably only related to the turnover of PPases and is therefore slower. For example, free MCs in *D. polymorpha* exposed for 21 days to *P. agardhii* (1 µg MC/L) were eliminated from tissues after 3 days in clean water, while total MCs were still detected after 21 days [26]. However, potentially because of *Anodonta* sp.’s less efficient detoxification system compared to that of *D. polymorpha* [62], free and total MCs in the digestive glands of *A. anatina* were still detected within tissues after 21 days in clean water.

Thus, free MCs—especially in *D. polymorpha*—may more rapidly reflect the dynamics of intracellular MC concentrations in the environment. In contrast, since total MCs (i.e., free plus bound forms) persist longer in tissues, their presence in caged bivalves may reflect either current or past water contamination by MC-producing cyanobacteria. This could explain why the density of MC-producing cyanobacteria was correlated with free MC concentrations in whole *D. polymorpha* in Lake Ailette but not with free MC concentrations in the digestive glands of *A. anatina*, nor with total MC concentrations in either species. Therefore, total MC concentrations in bivalves may not be appropriate for assessing short-term fluctuations in the presence or absence of MC-producing cyanobacteria in water.

However, the analysis of total MCs may be more suitable for ecotoxicological studies or health risk assessments, as it reflects the cumulative contamination experienced by organisms and may also help detect low MC concentrations indicative of the early stages of a bloom. One way to avoid overestimating the actual risk when using total MCs as a marker could be to regularly renew caged bivalves during the monitoring program.

In Lake Der, the accumulation of MCs in *A. anatina* tissues showed a strong correlation with both the total cyanobacterial density and the biovolume of MC-producing cyanobacteria. This relationship became particularly significant when intracellular MC concentrations exceeded 1 µg/L, indicating that bivalves are sensitive indicators of toxin presence and accumulation. Moreover, the frequency of MC-positive samples was higher in mussel tissues than in water samples, particularly at the Giffaumont and Chevalier sites. For instance, at Giffaumont, four out of five mussel samples tested positive for MCs, compared to only two out of five water samples. These findings confirm the role of bivalves as biological sentinels, with their tissues providing a time-integrated view of MC contamination events that might be missed by sporadic water sampling.

Using bivalves as biological indicators has the following advantages:

- It provides a more accurate understanding of overall lake contamination by MCs. Unlike instantaneous water quality measurements, bivalves offer temporal integration of contamination, enabling more reliable trend detection. Their ability to filter large volumes of water makes them highly sensitive to low MC concentrations, particularly when contamination is sporadic or localized, as demonstrated in this study.
- It offers insight into the potential transfer of MCs to higher trophic levels, including terrestrial food webs, since bivalves represent a food resource for many animals.
- It provides an indirect indication of contamination in aquatic products consumed by humans. Indeed, monitoring MCs in fish remains rare and challenging due to the difficulty of collecting sufficient individuals from target species for a single analysis. In contrast, total MC quantification has been successfully performed in mussel tissues but may be more difficult in fish muscle matrices, despite the potential toxicity of protein-bound MCs for consumers [10]. Further studies could then be conducted to establish the relationship between mussel and some fish contamination by free and total MCs during cyanobacterial blooms. In addition to environmental monitoring, the use of freshwater bivalves as bioindicators must also consider their physiological responses to toxic cyanobacteria. Indeed, MC-producing cyanobacteria can negatively affect bivalve growth, condition, and overall health, especially under prolonged or repeated exposure. These sublethal effects, recently reviewed [63], highlight the dual role of mussels as both sentinels and sensitive organisms exposed to cyanotoxins, reinforcing the relevance of integrating biological responses in future biomonitoring strategies.

## 4. Conclusions

We investigated in situ the potential use of various environmental parameters, along with the freshwater mussels *A. anatina* and *D. polymorpha* as integrative tools to biomonitor the presence of MC-producing cyanobacteria in three moderately contaminated lakes. Results showed that phycocyanin (PC) concentration, as well as the biovolume of total or MC-producing cyanobacteria, can serve as reliable predictors of MC presence in the environment—but only when biovolumes exceed 40 mm^3^/L. 

Our study also demonstrated that both mussel species accumulated free and total MCs even when intracellular MC concentrations in water remained below 1 µg/L. The use of bivalves as bioindicators of microcystins (MCs) is therefore relevant and effective. Their bioaccumulation capacity allows for continuous monitoring of toxic cyanobacterial contamination. This biomonitoring approach complements conventional water analyses and represents a valuable tool for the management and preservation of aquatic ecosystems. In addition, understanding the link between MC accumulation in mussels and in fish would help to further consolidate the role of bivalves as freshwater sentinels and to assess potential risks to human and ecological health.

## 5. Materials and Methods

### 5.1. Biological Materials

*Anodonta anatina* individuals (average shell length: 6.5 ± 1 cm), obtained from Univers aquatique (Sartrouville, France), were acclimated in groups of 50 in 15 L aerated aquaria filled with Cristalline^®^ spring water (Saint Yorre, France) maintained at 14 ± 2 °C under a controlled 12 h light/12 h dark photoperiod. During the acclimation phase, mussels were fed twice weekly with *Chlorella vulgaris* at a dose of 3.7 × 10^7^ cells per individual per day (Greensea, Mèze, France). Algal cell concentrations were determined using an optical microscope (Primovert, Zeiss, Oberkochen, Germany) and KOVA^®^ counting chambers (VWR, Fontenay-sous-Bois, France). Specimens of *Dreissena polymorpha* were sampled from Lake Der-Chantecoq (coordinates: 48°36′07.7″ N; 4°44′37.0″ E) and their acclimation procedures followed the protocol described in [64]. Considering the influence of shell size on filtration efficiency, individuals were selected to ensure uniformity in size (2 ± 0.3 cm).

### 5.2. Site Characterization and Caging

**Ailette**

**Figure 9:**
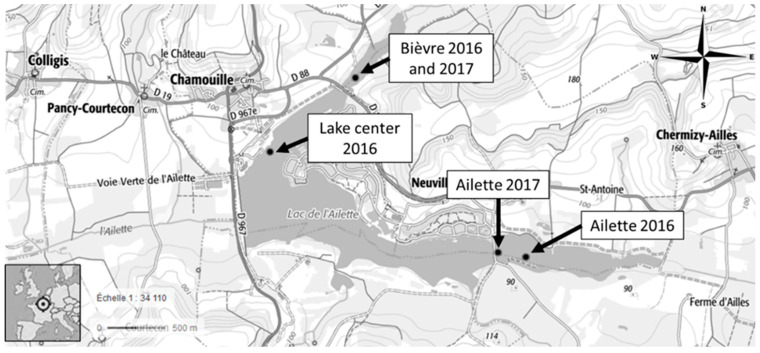
Location of the sampling sites in Ailette Lake with additional information regarding the year in which the study took place.

The Ailette lake is a large (132 Ha) and shallow (mean depth of 2 m) artificial reservoir located in France. It possesses two upstream stagnant lake tails: the Ailette (24.3 Ha, mean depth of 2 m) and the Bièvre (87.4 Ha, mean depth of 1 m), respectively fed by the Ailette and Bièvre rivers (Figure 9). Until 2018, during the dry season both tails were hydrologically isolated from the main part of the lake, promoting water stagnation. Both tails are also undergoing important fishing activities, a practice known to enhance eutrophication [35,36]. Fishing is prohibited in the main lake. An important cyanobacterial bloom associated with major fish mortality (500 kg of fishes) occurred in 2015 in the Ailette tail.

Prior to this study, we ensured that *D. polymorpha* was present in all sites. *D. polymorpha* and *A. anatina* were distributed in 3 mm-mesh polyethylene cages (7 × 18 cm) in groups of 16 and 5 mussels, respectively. Both sampling campaigns included the summer period, corresponding to cyanobacteria development in fresh waters. In addition, to study MC accumulation by mussels during both sampling years (2016 and 2017), the cages were immersed at a similar depth (1 m). Complementarily to mussel sampling, the water was sampled at the same depth as the cages to determine intracellular MC concentrations in the phytoplankton biomass and analyze the composition of the phytoplankton community.

In 2016, the study sites were in three locations: the Ailette tail (“Ailette”, 49°27′39.6″ N 3°41′56.5″ E), the Bièvre tail (“Bievre”, 49°28′29.5″ N 3°40′45.8″ E), and in the main lake (“lake center”, 49°28′03.5″ N 3°40′03.5″ E). For reasons of site accessibility, sampling occurred in July, August, and September in the Ailette and lake center sites and from August to November in the Bièvre site. In 2017, cages of *A. anatina* and of *D. polymorpha* were placed in July in Bièvre (the same location as 2016) and Ailette (49°27′41.5″ N 3°41′48.6″ E). Sampling occurred weekly in August.

**Lake Der-Chantecoq**

**Figure 10:**
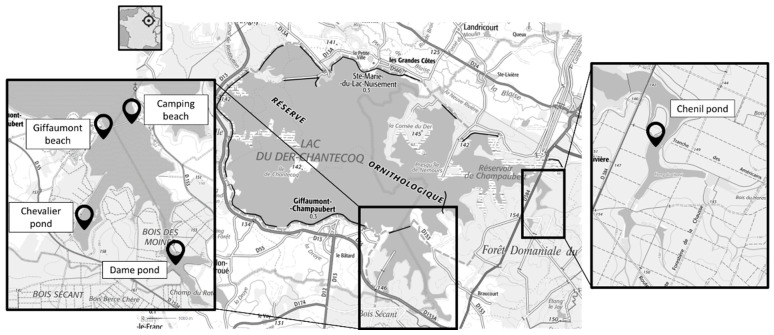
Location of the sampling sites in the Der-Chantecoq Lake. **“Réserve ornithologique” means** "bird reserve".

The Lake Der-Chantecoq (4800 Ha, maximum depth: 18 m, average depth: 4 to 7 m) is a large lake where many activities have developed (i.e., fishing, sailing, and swimming). The lake is divided into 4 basins: the old Champaubert reservoir, the main basin, and two delayed emptying basins (north and south nautical basins) isolated by submersible dikes and designed for tourist facilities (Figure 10). The monitoring occurred during the cyanobacteria proliferation season, with dates that varied depending on the site due to accessibility issues.

In the southern basin, two stations were studied from June to October 2017 and from April to October 2018 (Chenil and the camping beach) and three stations were studied from September to October 2017 and from April to October 2018 (Giffaumont, Chevalier, and La Dame). The stations at Giffaumont beach (“Giffaumont”, 48°33′05.2″ N 4°46′39.8″ E) and the camping beach (“camping”, 48°33′14.722″ N 4°47′34.61″ E), located in the basin, were partially connected to the lake during the high-water period. Old ponds and tails of the lake were also monitored, located in the eastern part and separated from the main basins by non-submersible dikes equipped with drainage works which allow water to be maintained during the descent from lake level. These stations in the Dame pond (“Dame”, 48°32′00.2″ N 4°47′48.3″ E), which was hyper eutrophic, in connection with the excessive nitrogen and phosphorus inputs by the Braucourt River and an accumulation of organic sludge, and the Chevalier pond (“Chevalier”, 48°32′20.6″ N 4°46′40.4″ E), were only supplied by their own watershed.

Further north, one station was studied monthly from June to October 2017 and from April to October 2018. This was the Chenil pond (area 0.1 km^2^, “Chenil”, 48°33′50.9″ N 4°50′55.8″ E), a former eutrophic fish-farming pond fed by non-permanent water bodies and several forest streams (Figure 10).

**Lake Temple**

**Figure 11:**
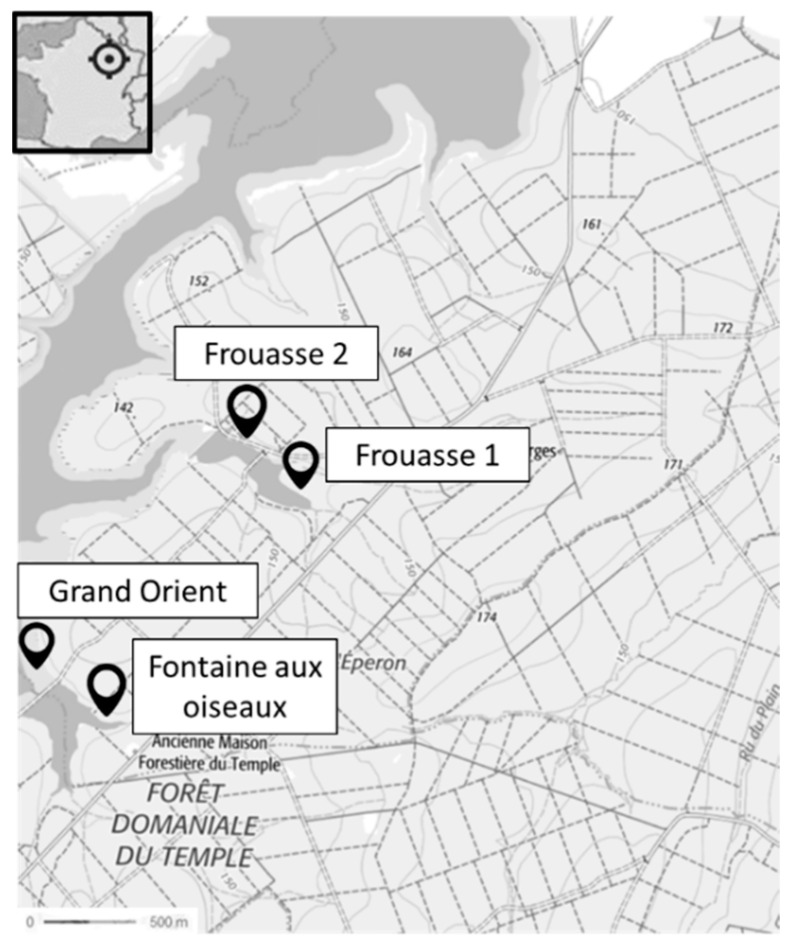
Location of the sampling sites in Temple Lake. *“Forêt domaniale du Temple”* means “State-owned Forest of Le Temple”.

This lake has an area of 2320 Ha (maximum depth: 11 m, mean depth: 5–6 m). Several activities are practiced there, including sailing, swimming, and fishing. At the Temple Lake, the Fontaine aux Oiseaux (“Fontaine aux Oiseaux”, 48°18′08.9″ N 4°26′25.0″ E) retaining tails (drained every 5 to 10 years), Frouasse 1 (“Frouasse 1”, 48°18′57.7″ N 4°27′01.5″ E) (always kept in water), and Frouasse 2 (“Frouasse 2”, 48°18′54.8″ N 4°27′13.3″ E) were studied from June to October 2017 and from April to October 2018, then the Grand Orient station (“Grand Orient”, 48°18′14.0″ N 4°26′10.2″ E) was studied in September 2017 and from April to October 2018 (Figure 11).

### 5.3. Analysis of Water Samples and Bivalve Tissues

The Chl a, the PC, the composition of the phytoplankton community, the cyanobacterial density and biovolume, and the MC-producing cyanobacterial density and biovolume were measured in all of the sites (see Section 5.3.2). In the Der and Temple Lakes, other environmental parameters were measured (temperature; pH; dissolved O_2_; total P; total N; conductivity; and SOM) and provided by the Seine Great Lakes Public Territorial Basin Establishment. At the end of each exposure period, mussels were retrieved from the cages at each site. Both years, upon their collection, the animals were transported in 1 L bottles containing lake water. They were then dissected and stored at −80 °C. As previously detailed [29], we collected the digestive gland of *A. anatina* and the whole body of *D. polymorpha*. Then tissues were freeze dried and grinded. Because of its large size, *A. anatina* was studied individually (n = 5 digestive glands in 2016, n = 4 in 2017), whereas the digestive glands of *D. polymorpha*, a smaller species, were pooled (n = 3 pools of 3 individuals in 2016 and 4 pools of 6 mussels in 2017). Each year, prior to caging, we checked the absence of free MCs in 5 digestive glands of *A. anatina* and 3 pools of 3 *D. polymorpha*.

#### 5.3.1. Measurement of Free and Total MCs in Phytoplankton Biomass and Bivalves

Water samples of 500 mL per site were filtered using 1 µm cyclopore track etched membrane to collect phytoplankton biomasses (Whatman, Maidstone, UK). MCs were extracted from the biomasses released in filters as described in [26]. To extract free microcystins (MCs) from mussel tissues, 10 mg of freeze-dried material was mixed with 1 mL of 80% methanol (MeOH). The mixture was first placed in an ultrasonic water bath (35 kHz) for 15 min, then processed through three successive rounds of centrifugation and sonication using a UP200S probe sonicator (Hielscher Ultrasonics GmbH, Teltow, Germany). Each sonication lasted 1 min at 30% amplitude and 50% duty cycle, followed by centrifugation for 10 min at 2500 rpm. The resulting supernatant was stored at 4 °C. A second extraction was performed on the remaining pellet using 1 mL of fresh 80% MeOH, following the same procedure. Both extracts were then combined and preserved at –80 °C prior to analysis. Quantification of free MCs was carried out in duplicate at different dilution levels (1:10, 1:50, and 1:100) using enzyme-linked immunosorbent assay (ELISA) kits (Abraxis LLC, Warminster, PA, USA), following the manufacturer’s instructions. Absorbance readings at 450 nm were obtained with a microplate reader (Tecan, Männedorf, Switzerland). Results were expressed as micrograms of MC per gram of dry tissue weight (µg MC/g DW).

The determination of total MC content was performed according to [26]. In brief, freeze-dried mussel tissues were transferred into glass tubes containing 0.1 M Sörensen phosphate buffer at pH 7.5. Samples were enzymatically hydrolyzed for two hours at 37 °C using a trypsin–EDTA mixture under constant agitation. Following hydrolysis, chemical oxidation was carried out for three hours at 37 °C using a 0.025 M solution of KMnO_4_/NaIO_4_ (pH 9), with gentle stirring throughout. The oxidative reaction was then halted by adding 40% sodium bisulfite until the solution became clear. The pH was subsequently checked and then adjusted with 10% sulfuric acid (*w*/*w*) if above 3.

Extracts were purified using 3 cc solid-phase extraction (SPE) cartridges (60 mg, Waters, Milford, MA, USA), preconditioned with 5 mL of ultrapure water followed by 5 mL of absolute methanol. After sample loading, cartridges were sequentially washed with 5 mL of water and 5 mL of 15% methanol, then dried. Elution was performed with 2 mL of 80% methanol and the eluates were evaporated to dryness under a nitrogen stream at 35 °C. The residues were then reconstituted in 200 µL of 35% methanol, centrifuged, and stored at 4 °C prior to analysis.

Quantification of the released MMPB (2-methyl-3-methoxy-4-phenylbutyric acid) was conducted by LC-MS/MS using a Waters Synapt mass spectrometer paired with an Alliance e2695 HPLC system (Waters, Milford, MA, USA), managed with MassLynx software (version 4.1, Micromass, Manchester, UK). Chromatographic separation was achieved on an Agilent ZORBAX SB-C18 column (5 µm, 3.0 × 250 mm, Agilent Technologies, Inc., Santa Clara, CA, USA) maintained at 30 °C. The mobile phase consisted of solvent A (0.1% formic acid in water) and solvent B (methanol), with a gradient ramping from 25% to 90% B over 12 min, followed by a 2 min hold at 90%, at a flow rate of 0.3 mL/min. Injections of 10 µL were analyzed using positive electrospray ionization in multiple reaction monitoring (MRM) mode. The monitored transitions were *m*/*z* 209.2 → 131.1 and 209.2 → 91, with the source parameters set as follows: desolvation gas flow 800 L/h, capillary voltage 4 kV, and cone voltage 5 V.

Total MC quantification was carried out using an external calibration approach adapted to the mussel tissue matrix. Calibration curves were established by fortifying unexposed tissues (n = 3 for each species) with a series of nine MMPB concentrations. After extraction and reconstitution in 35% methanol as previously described, samples were enriched with 2, 4, 8, 10, 12, 50, 150, 300, and 800 ng of MMPB to construct the standard curve. To assess the extraction efficiency for total MCs from either whole *D. polymorpha* or *A. anatina* digestive glands, additional tissue samples from non-contaminated individuals were spiked with MC-LR at three levels—1, 100, and 1000 µg—prior to the oxidation step.

As already reported [26], the average recovery and detection rates for total MCs from freeze-dried tissues varied depending on the spiked concentration range. For *A. anatina* digestive glands, recovery ranged from 32.9 ± 0.02% to 58.8 ± 16.1%, while for whole *D. polymorpha* specimens, values ranged from 36.5 ± 1.2% to 67.0 ± 14.9%. To obtain accurate quantification, total MC concentrations were adjusted based on extraction efficiency corresponding to the concentration range. Final results were reported as micrograms of MC per gram of dry weight (µg/g DW), corrected for recovery.

#### 5.3.2. Identification and Quantification of Cyanobacteria

The identification of cyanobacterial genera and species, as well as the evaluation of their densities, were performed twice monthly. Water was sampled on the first meter with a bailer and transferred into 500 mL untreated plastic bottles. Cyanobacteria genera and species were identified, counted in a 50 μL Nageotte chamber, and density was expressed in cells/mL. The frequency of occurrence (FO) was calculated as the percentage of samplings in which each cyanobacteria species was present. Biovolumes of cyanobacteria were measured using the database made by the Centre d’expertise en analyse environnementale (CEAEQ, Quebec City, QC, Canada). PC and Chl a concentrations were evaluated using a Trios fluorometric probe (TriOS Optical Sensor, TriOS Mess- und Datentechnik GmbH, Rastede, Germany). For the Der and Temple Lakes, we focused our study only on cyanobacteria populations.

### 5.4. Statistics

All statistical analyses were conducted using Statistica software (Version 8, StatSoft, Tulsa, OK, USA, 2007). The distribution normality of datasets was assessed using the Shapiro–Wilk test, while variance homogeneity was evaluated through Levene’s test. As both tests yielded *p*-values below 0.01, indicating non-normality and unequal variances, non-parametric methods were applied. Specifically, group comparisons across multiple independent datasets were performed using Kruskal–Wallis tests, and pairwise comparisons relied on the Mann–Whitney U test. Relationships between variables were explored using Pearson correlation coefficients, with statistical significance defined at *p* < 0.05. To explore patterns and associations among environmental and cyanobacterial variables, a Principal Component Analysis (PCA) was carried out using the STATISTICA for Windows software package (version 6.0, StatSoft Inc., Tulsa, OK, USA).

## Data Availability

The original contributions presented in this study are included in this article and Supplementary Materials. Further inquiries can be directed to the corresponding author.

## Supplementary Materials

The following supporting information can be downloaded at: https://www.mdpi.com/article/10.3390/toxins17050245/s1, Figure S1: Proportional abundance of phytoplankton taxonomical classes in the Bièvre site BI (a), Lake Center LC (b) and Ailette site AI (c) of the Ailette Lake, between May and August 2016; Figure S2: Proportional abundance of phytoplankton taxonomical classes in the sites Bièvre (BI, a) and Ailette (Al, b) of the Ailette Lake in August 2017; Table S1: Environmental and cyanobacterial parameters measured at various sites of the Ailette, Der, and Temple lakes during 2017 and 2018 field campaigns. The table includes data on MC-producing cyanobacteria (cell density and biovolume), total cyanobacterial biovolume, air and water temperature, rainfall, pH, dissolved oxygen, oxygen saturation, biochemical and carbonaceous oxygen demand, orthophosphates, total phosphorus, various nitrogen forms (ammonium, nitrites, nitrates, total nitrogen), silica, chlorophyll a, conductivity, turbidity, total suspended matter (TSM), and phycocyanin.
