# Supplementary material for: Survey of Microcystin-Producing Cyanobacteria in French Lakes of Various Trophic Status Using Environmental and Cyanobacterial Parameters and an Active Mussel Biomonitoring
Source: Toxins (Basel). 2025 May 15;17(5):245. doi: 10.3390/toxins17050245 (PMC12115459; doi:10.3390/toxins17050245)
Supplement: Supplementary file 1 [file toxins-17-00245-s001.zip › Supplementary Material.pdf]

Supplementary Material Figure S1: Proportional abundance of phytoplankton taxonomical classes in the Bièvre site BI (a), Lake Center LC (b) and Ailette site AI (c) of the Ailette Lake, between May and August 2016

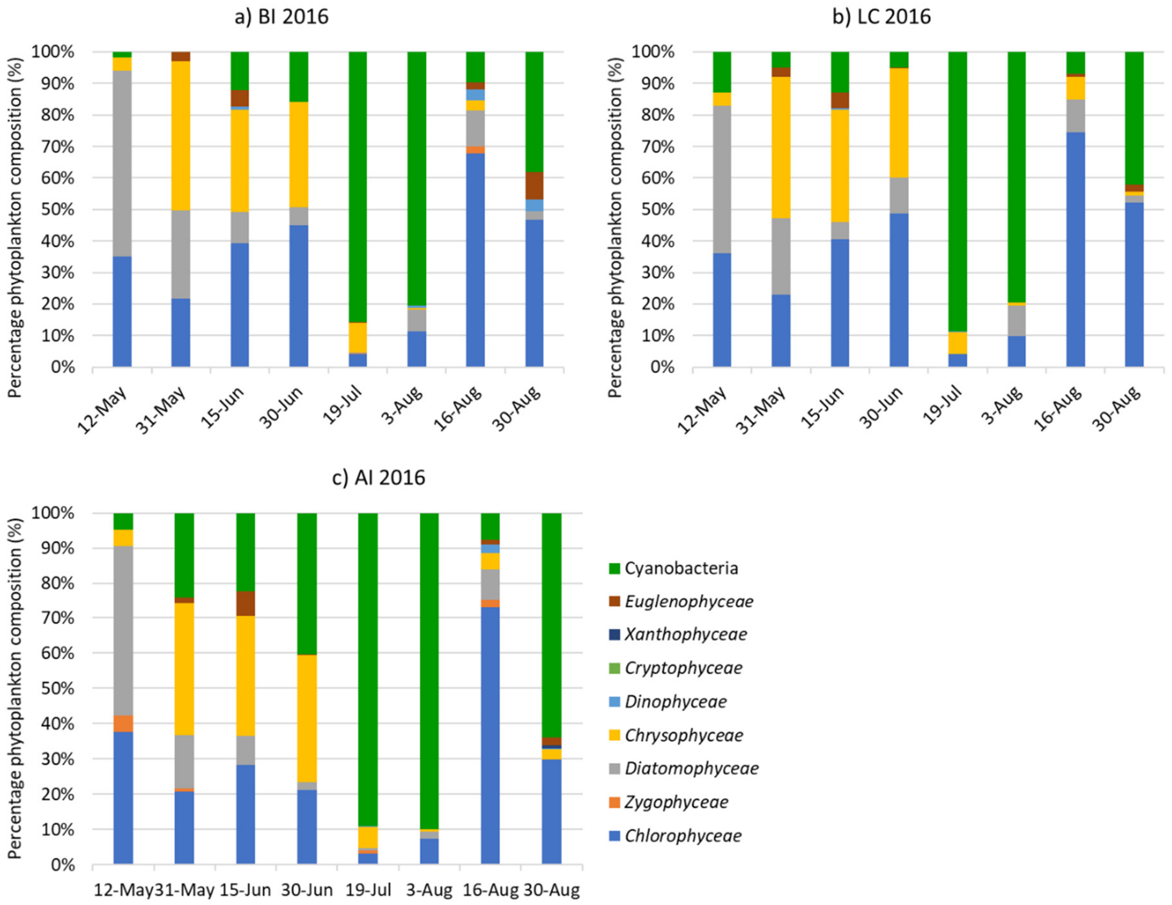

Supplementary Material Figure S2 : Proportional abundance of phytoplankton taxonomical classes in the sites Bièvre (BI, a) and Ailette (AI, b) of the Ailette Lake in August 2017.

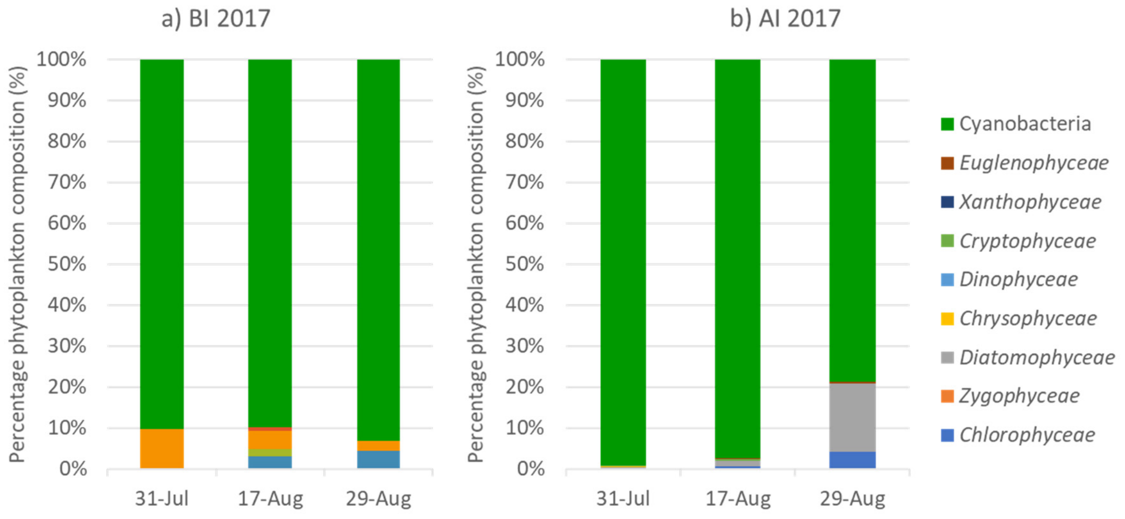

Supplementary Material Table S1 – Environmental and cyanobacterial parameters measured at various sites of the Ailette, Der, and Temple lakes during 2017 and 2018 field campaigns.

The table includes data on MC-producing cyanobacteria (cell density and biovolume), total cyanobacterial biovolume, air and water temperature, rainfall, pH, dissolved oxygen, oxygen saturation, biochemical and carbonaceous oxygen demand, orthophosphates, total phosphorus, various nitrogen forms (ammonium, nitrites, nitrates, total nitrogen), silica, chlorophyll a, conductivity, turbidity, total suspended matter (TSM), and phycocyanin.

Please see excel file attached

Site	Lake	MC-prod cyano (cell/ml)	Biovolume cyano	MC-prod cyano biovolume	Air temprature °C	Rainfall	Water temprature (°C)	pH	Dissolved oxygene (mg O2 /L)	Oxygen saturation rate (%)	Biochemical oxygen demand (mg O2 /L)
Chenil	Der	97920	3.413797	1.109797	32	0	29.7	10	13.4	176.2	0
Chenil	Der	1940160	151.2016	151.07616	10	0.2	13.7	8	11.8	111.2	42
Chenil	Der				25	0	23.6	8.82	9.5	113.1	3
Chenil	Der	64766	8.24	8.24	25	0	23.5	8.57	12	141.1	1
Chenil	Der	1776000	115.48	114.4432	25	0	23.4	9.95	15.4	182.7	0
Chenil	Der	1294368	131.57	131.57	28	0	20.8	7.7	7.7	85.9	27
Chenil	Der	0	27.21	0	20	0	17.5	7.4	8.02	82.5	23
Chenil	Der	0	0	0	7	2	6.6	7.7	9.9	80.8	
Chenil	Der	143520	12.52	12.52	12	0	13.2	8.4	12.2	116.4	17
Plage Camping	Der				25	0	21.6	8.29	11.8	134.4	0
Plage Camping	Der	720	3.08	3.08	25	0	23.2	8.1	10.26	120.3	3
Plage Camping	Der	0	0	0	25	0	23.7	8.43	11.4	135.4	6
Plage Camping	Der	0	0.29	0	28	0	23.5	8.5	8.32	98	3
Plage Camping	Der	36600	13.45	13.4212	20	0	19.1	9.13	13	140.4	10
Plage Camping	Der	1224	1.166376	1.160616	12	0	14.7	8.51	10.14	99.8	5
Giffaumont	Der				25	0	25	8.69	11.5	135.5	1
Giffaumont	Der	0	0	0	25	0	22.4	7.71	9.6	110.7	2
Giffaumont	Der	0	0	0	25	0	25.6	8.21	9.54	117.1	4
Giffaumont	Der	164	0.35	0.007776	28	0	23.5	8.5	8.32	98	3
Giffaumont	Der	522	1.2	0.006264	22	0	20.2	8.42	9.54	105.3	3
Giffaumont	Der	39696	12.911636	12.902996	21	0	21	8.6	9.8	109.8	38
Giffaumont	Der	0	0	0							
Giffaumont	Der	0	0.19	0	12	0	15	8.66	11.9	117.6	6
Etang Chevalier	Der				25	0	22.4	8.42	9.86	113.1	0
Etang Chevalier	Der	0	0.33	0	25	0	22.3	8.25	10.5	120.7	0
Etang Chevalier	Der	1920	8.21	8.21	25	0	24.5	8.11	9.12	109.7	0
Etang Chevalier	Der	3120	13.34	13.34	28	0	23	7.8	7.56	88.1	3
Etang Chevalier	Der	1712	0.02	0.02	22	0	18.7	7.86	7.42	79.4	3
Etang Chevalier	Der	2266	0.06	0.027192	12	0	14.8	8.3	10.3	101.5	5
Etang la Dame	Der				25	0	23.6	8.97	10.9	128.8	6
Etang la Dame	Der	36160	54.45	54.45	25	0	23.4	8.47	12.6	147.9	2
Etang la Dame	Der	4608	19.7	19.6992	25	0	25	8.82	12.67	154.2	6
Etang la Dame	Der	6326400	747.78	747.78	28	0	22.9	9.2	10.6	123	1
Etang la Dame	Der	0	207.13	0	22	0	18.6	8.81	10.8	115.3	34
Etang la Dame	Der	1320	0.368208	0.139752	12	0	14	8.77	9.6	93.5	13
Etang Dame	Der	181600	160.59456	160.39296	25	0.2	15.3	8.98	15.3	153	0
Etang Dame	Der	0	0.6704	0							
Fontaine aux oiseaux	Temple	0	0.0072	0	21	0	24.3	8.5	9.4	112.7	5
Fontaine aux oiseaux	Temple	2	0.00001	0.00001	20	0	15	7.7	9.3	92.1	0
Fontaine aux oiseaux	Temple	0	0.004032	0	20	0	15.8	7.34	8.8	86.5	7
Fontaine aux oiseaux	Temple	0	0	0							
Fontaine aux oiseaux	Temple				25	35	23.5	7.16	8.4	98.7	6
Fontaine aux oiseaux	Temple	72	0.45	0.3078	10	0	23	7.49	9.8	112.7	5
Fontaine aux oiseaux	Temple	0	0	0	22	0	25	7.92	9.19	111.3	5
Fontaine aux oiseaux	Temple	1992	0.18	0.18	23	0	22.6	7.2	8.41	97.3	3
Fontaine aux oiseaux	Temple	384	0.28	0.28	12	0	17.6	7.6	7.3	76.7	2
Fontaine aux oiseaux	Temple	0	0	0	12	0	14.7	8.05	9.05	89.01	0
Frouasse 1	Temple				25	35	23	7.75	10.62	124.1	7
Frouasse 1	Temple	1200	5.15	5.13	10	0	22	7.6	7.83	89.3	7
Frouasse 1	Temple	0	0	0	22	0	24.6	7.21	5.3	62.3	8
Frouasse 1	Temple	3648	0.32	0.32	21.5	0	21.5	7.2	8.02	91.7	21
Frouasse 1	Temple	0	0.01	0	12	0	14.2	7.8	8.52	82.7	5
Frouasse 2	Temple				25	35	23.2	7.6	7.7	89.1	6
Frouasse 2	Temple	3840	16.58	16.416	10	0	22	7.14	7.9	90.2	6
Frouasse 2	Temple	0	0	0	22	0	24.5	7.21	8.62	103.3	6
Frouasse 2	Temple	3312	0.29	0.29	23	0	22	8.1	8.02	91.7	4
Frouasse 2	Temple	893	0.08	0.08	12	0	17.5	7.61	7.8	79.6	4
Frouasse 2	Temple	0	0.04	0	12	0	14	7.7	8.49	82.4	6
Frouasse 2	Temple	2400	0.0216	0.01728	21	0	22.5	8.52	10.02	117.5	16
Frouasse 2	Temple	1080	0.304488	0.16308	15.3	0	15.3	8.35	11.4	113.8	0
Frouasse 2	Temple	0	0	0	20	0	15.9	7.28	9.1	93	1
Gra Orient	Temple				25	35	22.7	7.48	10	94.8	3
Gra Orient	Temple	0	0.39	0	10	0	23.3	7.81	8.3	97.5	2
Gra Orient	Temple	1896	2.508504	2.508504	20	0	20	7.87	10.5	100.5	11
Gra Orient	Temple	0	0	0							
Gra Orient	Temple	0	0.02	0	22	0	24.3	7.61	8.6	102.2	0
Aillette	Aillette	1600	0.046944	0.011584							
Aillette	Aillette	2266	0.0139236	0.0139236							
Aillette	Aillette	1600	0.046944	0.011584							
Aillette	Aillette	2266	0.0139236	0.0139236							
Aillette	Aillette	974	0.097136	0.067718							
Centre	Aillette	0	0.268928	0.00000							
Centre	Aillette	108	0.00648768	0.00648768							
Centre	Aillette	600	0.0678	0.0678							
Centre	Aillette	0	0.268928	0							
Centre	Aillette	108	0.0678	0.0678							
Bièvre	Aillette	199	0.0112484	0.0077124							
Bièvre	Aillette	240	0.0074704	0.0074704							
Bièvre	Aillette	760	0.08028	0.07972							
Bièvre	Aillette	240	0.0074704	0.0074704							
Bièvre	Aillette	760	0.08028	0.07972							
Aillette	Aillette	310560	10.944096	9.507936							
Aillette	Aillette	108240	28.288572	26.349372							
Aillette	Aillette	108240	28.288572	26.349372							
Aillette	Aillette	31200	2.613024	1.456992							
Aillette	Aillette	310560	10.944096	9.507936							
Aillette	Aillette	108240	28.288572	26.349372							
Aillette	Aillette	108240	28.288572	26.349372							
Aillette	Aillette	31200	2.613024	1.456992							
Bièvre	Aillette	0	1.0944	0							
Bièvre	Aillette	7680	0.847488	0.012288							
Bièvre	Aillette	7680	0.847488	0.012288							
Bièvre	Aillette	0	0.3456	0							
Bièvre	Aillette	0	1.0944	0							
Bièvre	Aillette	7680	0.847488	0.012288							
Bièvre	Aillette	7680	0.847488	0.012288							
Bièvre	Aillette	0	0.3456	0							

Site	Carbonaceous oxygen demand (mgO2/l)	Orthophosphates (mg PO43- /L)	Phosphate maximal (mgP/L)	Phosphore total (mg P/L)	Ammonium (mg NH4+/L)	Nitrites (mg NO2- /L)	Nitrates (mg NO3- /L)	Total Nitrogen (mg N/L)	Silice (mg SiO2 /l)	Chl A (µg/l)	Conductivity (µS/cm)	Turbidity	Total Suspended Matter (MES)	Phycocyanin (µg/l)
Chenil	74.3	0.25	0.081566069	0.08	0.02	0.03	9	5	11	347	164	118	73.6	7.53
Chenil	74.3	0.05	0.016313214	0.02	0.08	0.18	7	5.4	14	394	158	110	79	151.31
Chenil	51.5	0.34	0.110929853	0.11	0.02	0.03	1	1.9	5	49	178	40	28.7	
Chenil	44.3	0.08	0.026101142	0.03	0.01	0.03	0	2.1	9	30	164	20	20.75	24.37
Chenil	73.8	0.08	0.026101142	0.03	0.03	0.05	0	11.7	14		151	125	205	>= 200
Chenil	68	0.23	0.01	0.08	0.08	0.06	0	7.9	2	13	234	103	95	116.09
Chenil	72.4	0.22	0.07177814	0.02	0.14	0.04	0	6.7	17	46	135	82	85	35.28
Chenil	34.8	0.26	0.084828711	0.24	0.45	0.24	30	8.7	8		291	39	28.5	0
Chenil	72.8	0.3	0.097879282	0.08	0.05	0.04	0	6	12	330	127	53	54.6	19.83
Plage Camping	13.1	0.02	0.006525285	0.01	0.08	0.11	8	2.3	1	14	422	2	2.8	
Plage Camping	5	0.04	0.013050571	0.01	0.03	0.12	7	2.1	0	11	316	4	2.8	0.59
Plage Camping	17.8	0.05	0.016313214	0.02	0.01	0.04	0.6	1.2	1		249	6	6.8	0
Plage Camping	30.2	0.03	0.009787928	0.01	0.24	0.04	1	1.7	5	11	234	10	5.2	
Plage Camping	37.2	0.05	0.016313214	0.02	0.14	0.06	0	2.5	5	26	195	20	16.25	18.55
Plage Camping	37.1	0.06	0.019575856	0.02	0.29	0.04	0.3	2.7	3	37.5	203	19	16.6	2.74
Giffaumont	15	0.04	0.013050571	0.01	0.06	0.07	3	1.7	1	25	279	8	0.4	
Giffaumont	14	0.05	0.016313214	0.02	0.03	0.08	2	1.5	2	15	255	4	1.4	1.47
Giffaumont	13.1	0.05	0.016313214	0.02	0.1	0.06	0.5	1.2	0		226	9	10.6	0.44
Giffaumont	30.2	0.03	0.009787928	0.01	0.24	0.04	1	1.7	5	11	234	10	5.2	1.9
Giffaumont	14	0.06	0.019575856	0.02	0.1	0.03	0	1.1	5	13	223	9	9.4	2.88
Giffaumont	75.8	0.01	0.003262643	0.01	0.04	0.04	3	4.8	6	309	208	170	60	14.02
Giffaumont														0
Giffaumont	20.3	0.05	0.016313214	0.02	0.01	0.03	0.1	1.5	2	36	230	8	11.4	1.95
Etang Chevalier	14.9	0.02	0.006525285	0.01	0.03	0.06	3	1.8	1	22	308	2	0	
Etang Chevalier	14.5	0.04	0.013050571	0.02	0.02	0.09	2	1.5	3	16	256	6	3.6	
Etang Chevalier	13.1	0.05	0.016313214	0.02	0.06	0.04	0.4	1.3	0		223	7	6.8	0.24
Etang Chevalier	68.6	0.29	0.094616639	0.1	0.02	0.03	0	3.5	2	10	215	7.6	4.4	0.2
Etang Chevalier	15.4	0.07	0.022838499	0.02	0.14	0.03	0	1.6	5	19	227	11	18	2.34
Etang Chevalier	15.5	0.05	0.016313214	0.02	0.01	0.02	0.1	1.4	2	27	234	8	8.2	3.76
Etang la Dame	37.3	0.05	0.016313214	0.02	0.06	0.02	1	2.4	1	46	258	16	20.6	
Etang la Dame	35.8	0.06	0.019575856	0.02	0.01	0.02	0	1.5	1	73	236	14	14	9.38
Etang la Dame	33.5	0.06	0.019575856	0.02	0.01	0.03	0	1.2	0		223	12	19	3.61
Etang la Dame	72.1	0.04	0.013050571	0.01	0.02	0.04	0	1.2	1	65	171	351	246.3	>= 200
Etang la Dame	74.7	0.08	0.026101142	0.02	0.3	0.08	0	6.9	9	80	194	80	59.5	>= 200
Etang la Dame	61.1	0.1	0.032626427	0.03	0.15	0.05	0	3.9	1	36	208	26	29.6	8.55
Etang Dame	74.8	0.04	0.013050571	0.01	0.02	0.03	1	4.2	7	367	206	122	64	145.64
Etang Dame														0.15
Fontaine aux oiseaux	31.4	0.02	0.006525285	0.01	0.02	0.02	2	0.8	5	3	151	3	3.4	
Fontaine aux oiseaux	35.4	0.01		0	0.02	0.03	0	0.9	2	7	163	4	1.6	0.49
Fontaine aux oiseaux	26.8	0.05	0	0.02	0.05	0.03	0	1	4	4	172	3	1	
Fontaine aux oiseaux														0
Fontaine aux oiseaux	41.4	0.05	0.016313214	0.02	0.08	0.05	3	1.9	4	5	101	17	6.2	
Fontaine aux oiseaux	40.4	0.03	0.009787928	0.01	0.02	0.03	2	1.1	0	12	106	9	2.8	1.95
Fontaine aux oiseaux	34.4	0.05	0.016313214	0.02	0.02	0.04	0	1.2	5		122	5	3	0
Fontaine aux oiseaux	48.1	0.04	0.013050571	0.01	0.02	0.04	0	4.9	4	15	133	13	8.4	2.05
Fontaine aux oiseaux	38.1	0.06	0.019575856	0.02	0.12	0.03	0	2.3	7	22	182	63	8.75	0.59
Fontaine aux oiseaux	40.7	0.04	0.013050571	0.01	0.03	0.01	0	1.9	5	25	152	12	12	3.61
Frouasse 1	59.7	0.19	0.061990212	0.06	0.04	0.05	3	2.4	5	91	66	19		
Frouasse 1	50.5	0.17	0.055464927	0.06	0.02	0.05	2	0.8	0	13	111	14	14	24.37
Frouasse 1	47.2	0.13	0.042414356	0.04	0.01	0.04	0	1.6	0		135	7	10.4	0
Frouasse 1	74.1	0.04	0.013050571	0.01	0.02	0.06	0	7.3	2	87	74	25	14.6	7.67
Frouasse 1	69.9	0.18	0.058727569	0.06	0.9	0.08	0	4.2	5	17	83.1	24	15.6	4.69
Frouasse 2	43.9	0.12	0.039151713	0.04	0.03	0.05	3	1.6	5	11	93	26	13.4	
Frouasse 2	49.7	0.1	0.032626427	0.03	0.02	0.04	3	2.1	2	20	74	14	9.6	13.68
Frouasse 2	42.9	0.09	0.029363785	0.03	0.02	0.05	0	1.3	6		79	8	4.8	0
Frouasse 2	48.1	0.13	0.042414356	0.05	0.09	0.05	0	7.8	6	24	151	24.4	12.3	8.64
Frouasse 2	47.3	0.1	0.032626427	0.03	0.45	0.06	0	3.6	3	15	185	61	35.25	1.03
Frouasse 2	58	0.11	0.03588907	0.04	1	0.08	0	3.8	0	36	188	112	160	0
Frouasse 2	55	0.37	0.120717781	0.12	0.03	0.04	3	0.5	3	1	104	5	1.8	1.61
Frouasse 2	44.5	0.06	0.019575856	0.02	0.02	0.03	1	1.5	1	66	202	63	31.6	7.57
Frouasse 2	38.8	0.03	0.009787928	0.01	0	0.02	0	2.1	3	27	224	17	11.6	0.05
Gra Orient	34.1	0.09	0.029363785	0.03	0.23	0.06	6	1.6	4	16	118	16	5.4	
Gra Orient	12.9	0.03	0.009787928	0.01	0.01	0.08	3	1.1	0	18	280	3	2.6	2.58
Gra Orient	64.5	0.1	0.032626427	0.03	0.06	0.05	1	2.5	3	120	174	138	55.2	5.08
Gra Orient														0
Gra Orient	32.6	0.1	0.032626427	0.03	0.01	0.04	0	1.2	1		161	7	5.8	0.24
Aillette										32.1				3.2
Aillette										5.27				2.34
Aillette										32.1				3.2
Aillette										5.27				2.34
Aillette										7.8				3.7
Centre										3.3				2
Centre										1.86				2.2
Centre										3.7				2.5
Centre										3.3				2
Centre										1.86				2.2
Bièvre										13.6				5.09
Bièvre										2.34				2.15
Bièvre										11.92				5.71
Bièvre										2.34				2.15
Bièvre										11.92				5.71
Aillette										19.56				8.33
Aillette										20.85				14.21
Aillette										20.85				14.21
Aillette										24.71				12.36
Aillette										19.56				8.33
Aillette										20.85				14.21
Aillette										20.85				14.21
Aillette										24.71				12.36
Bièvre										17.76				5.31
Bièvre										33.94				3.81
Bièvre										33.94				3.81
Bièvre										7.03				1.61
Bièvre										17.76				5.31
Bièvre										33.94				3.81
Bièvre										33.94				3.81
Bièvre										7.03				1.61
